# Roots of Indian heliotrope (*Heliotropium indicum*) produce simple pyrrolizidine alkaloids using the same homospermidine oxidase involved in biosynthesis of complex pyrrolizidine alkaloids in aerial parts

**DOI:** 10.1111/plb.70077

**Published:** 2025-07-18

**Authors:** M. M. Zakaria, M.‐B. Salewski, D. Ober

**Affiliations:** ^1^ Botanical Institute and Botanic Gardens Kiel University Kiel Germany; ^2^ Department of Pharmacognosy, Faculty of Pharmacy Zagazig University Zagazig Egypt

**Keywords:** CRISPR/Cas9, homospermidine oxidase, Indian heliotrope, pathway evolution, specialized metabolism

## Abstract

- Pyrrolizidine alkaloids (PAs) are toxic specialized metabolites found in several plant lineages with independent evolutionary origins. In comfrey (*Symphytum officinale*), two independent homospermidine oxidase (HSO) paralogs are responsible for oxidation of homospermidine (Hspd) to the bicyclic pyrrolizidine in roots and young leaves. As PA biosynthesis in *S. officinale* and *Heliotropium indicum* (Indian heliotrope) share a common ancestor, we tested whether *H. indicum* is also able to synthesize PAs, not only in aerial parts but also in roots.
- *H. indicum* constitutively synthesizes not only complex PAs, in aerial parts but also simple PAs in roots. Of five copper‐containing amine oxidases (CuAOs) identified in *H. indicum*, three have the ability to convert Hspd to the bicyclic pyrrolizidine *in vitro*. CRISPR/Cas9 genome editing confirmed that, *in planta*, only one of these CuAOs is involved in PA biosynthesis in roots, which is identical to the HSO involved in PA biosynthesis in leaves.
- PA biosynthesis in roots is less efficient than in aerial parts, an observation that allowed the analysis of various pathway intermediates using tracer feeding experiments.
- The CuAO phylogeny, together with comparative gene structure analyses, suggest a common evolutionary origin of PA‐producing CuAOs. However, independent scenarios of PA metabolism were observed in Indian heliotrope and comfrey, adding a further aspect of diversity in the regulation of PA biosynthesis.

## Graphical abstract

Indian heliotrope uses the same oxidase to produce complex pyrrolizidine alkaloid esters in aerial parts and simple pyrrolizidine alkaloids in roots.

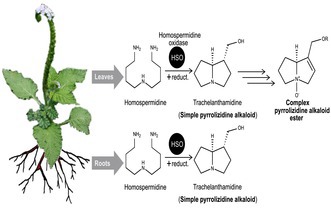

## INTRODUCTION

Plant secondary metabolism encompasses an enormous diversity of compounds and enzymes, as well as a wide range of mechanisms for gene regulation and secondary metabolite transport to cope with various abiotic and biotic stressors (Cordell 2013; Anjali *et al*. 2023). Many classes of plant secondary metabolites, also known as specialized metabolites or natural products, have a restricted occurrence only in specific organs, tissues, or cell types (Li *et al*. 2016), suggesting tight spatial regulation of the responsible biosynthetic pathways. For instance, plant defence metabolites are often produced in specialized tissues or cell types to avoid autotoxicity in the surrounding tissues (Schilmiller *et al*. 2010; Tissier 2012). In several cases, the sites of biosynthesis and accumulation differ. Examples are the alkaloids nicotine and hyoscyamine from *Nicotiana tabacum* and *Atropa belladonna*, respectively, which are synthesized in roots and transported to leaves to exert their defence functions in the tissue most likely to be attacked by herbivores (Hashimoto & Yamada 1994; Katoh *et al*. 2005; Tan *et al*. 2020). This has also been described for pyrrolizidine alkaloids (PAs) in *Senecio vulgaris*, where PAs are synthesized in roots and accumulate mainly in reproductive tissues of flower heads (Hartmann *et al*. 1989).

PAs are a typical class of secondary metabolites that serve as chemical defence against herbivores (Hartmann 1999; Facchini & St‐Pierre 2005; Florean *et al*. 2023). They have scattered occurrence in many distantly related angiosperms (Langel *et al*. 2011; Kaltenegger *et al*. 2013). In all PA‐producing lineages analysed so far, homospermidine synthase (HSS), the first pathway‐specific enzyme of PA biosynthesis, originates by duplication of the gene encoding deoxyhypusine synthase (DHS), an enzyme in primary metabolism (Ober & Hartmann 1999b). This duplication has occurred independently several times in the various PA‐producing lineages of angiosperms (Ober & Hartmann 1999a; Anke *et al*. 2004; Reimann *et al*. 2004; Nurhayati & Ober 2005; Kaltenegger *et al*. 2013; Irmer *et al*. 2015). HSS uses spermidine (Spd) and putrescine (Put) as substrates to catalyse the formation of homospermidine (Hspd), which is exclusively incorporated into the necine base moiety, the characteristic bicyclic ring skeleton of PAs (Böttcher *et al*. 1993, 1994; Zakaria *et al*. 2024). Hspd is oxidized at the two terminal amino groups by a PA‐specific copper‐containing amine oxidase (CuAO), homospermidine oxidase (HSO), to yield the bicyclic pyrrolizidine backbone (Zakaria *et al*. 2022, 2024).

Within the Boraginales, only one duplication event of the gene encoding HSS occurred early in the evolution of this lineage (Reimann *et al*. 2004). However, both the site of PA biosynthesis and the expression pattern of HSS have been shown to be diverse within this lineage. For example, in *Heliotropium indicum* (Indian heliotrope, Heliotropiaceae), HSS expression is found in the lower epidermis of the leaf and the stem epidermis (Niemüller *et al*. 2012), supporting previous studies by Frölich *et al*. (2007), who identified the shoot as the site of PA biosynthesis using tracer feeding experiments with radiolabeled putrescine. In *Symphytum officinale* (comfrey, Boraginaceae), PAs are constitutively produced in roots, with exclusive HSS expression in endodermis cells. In addition, a transient active site for PA biosynthesis has evolved in leaves subtending the developing inflorescences with HSS expression in the bundle sheath. The PAs are efficiently transported to the floral structures to boost PA levels in reproductive tissues (Kruse *et al*. 2017; Stegemann *et al*. 2019). Of note, in comfrey the gene encoding HSS, which catalyses the first step of PA biosynthesis, occurs as a single‐copy gene, while two independent HSO‐encoding paralogs are required for the subsequent Hspd oxidation at the two different PA‐producing sites of the plant: the root and young leaves (Zakaria *et al*. 2024). Frölich *et al*. (2007) reported the ability of *H. indicum* roots to synthesize Hspd after feeding with [^14^C]Put, which supports studies of Niemüller *et al*. (2012) using gene expression analyses, suggesting that HSS expression also occurs in roots of *H. indicum* as well as in PA‐producing aerial parts. These observations warrant a closer look at PA biosynthesis in the roots of this plant.

In this study, we show that, in addition to the previously identified site for complex PA esters in the aerial parts, Indian heliotrope has an additional site for biosynthesis of simple PAs in roots. The lower efficiency of the pathway in roots allowed a detailed analysis and inference of the accumulated pathway intermediates between Hspd and necine base formation using tracer feeding experiments. Using CRISPR/Cas9 genome editing, we further show that the gene, previously identified to drive PA biosynthesis in the aerial parts, is also involved in formation of the pyrrolizidine backbone in the roots, an aspect that we discuss in comparison to PA biosynthesis in comfrey, which uses two different paralogs at two distinct sites to produce complex PAs. The data show that despite the common origin of PA biosynthesis in these two plant species, different mechanisms of its regulation have evolved.

## MATERIAL AND METHODS

### Plant material and growth conditions

*Heliotropium indicum* and *Nicotiana benthamiana* seeds were grown in a climate chamber as previously described (Zakaria *et al*. 2022).

### Tracer feeding to roots of *H. indicum*

The [^13^C]Hspd was prepared enzymatically from [^13^C]Put (Eurisotop, France) using bacterial HSS and purified by ion exchange chromatography (Graser *et al*. 1998). The resulting [^13^C]Hspd and/or [^13^C]Put was fed to approximately 1 g of fresh root material from either 6‐month‐old hairy root (HR) containing the empty binary vector pB2GW7.0 (Karimi *et al.* 2007) or from 10‐week‐old wild‐type *H. indicum* plants. The 1‐formylpyrrolizidine substrate was prepared by *in vitro* incubation of affinity‐purified *Hi*CuAO1 (*Hi*HSO; Zakaria *et al*. 2022) with Hspd as described in Zakaria *et al*. (2022). The purified 1‐formylpyrrolizidine was applied to a liquid culture of HR *hicuao1*‐M1 with the inactivated *hicuao1* gene generated in this study for 15 days as described in Zakaria *et al*. (2021). HRs and roots of wild‐type plants were harvested at the end of the feeding experiments, washed with water, patted dry with tissue paper, and lyophilized for PA extraction and analysis as described in Zakaria *et al*. (2021).

### Relative transcript quantification in *H. indicum* roots

The RNA extraction, cDNA synthesis, and RT‐qPCR analysis of genes encoding HSS of *H. indicum* (*Hi*HSS) and the five recently identified genes encoding CuAOs of *H. indicum* (*Hi*CuAOs; Zakaria *et al*. 2022), *i.e*., *Hi*CuAO1, *Hi*CuAO2, *Hi*CuAO3, *Hi*CuAO4, and *Hi*CuAO5, were performed as previously described (Sievert *et al*. 2015) from 20‐week‐old HRs generated by infecting detached leaves of *H. indicum* with *Agrobacterium rhizogenes* carrying the empty pB2GW7.0 binary vector (Karimi *et al*. 2007), and from roots of 10‐week‐old wild‐type plants. RT‐qPCRs were performed in triplicate. The gene encoding ubiquitin was used as a reference gene. The primers used have been described previously (Zakaria *et al*. 2022).

### Generation of binary vector constructs and transient protein expression in *N. benthamiana*

The complete ORFs of *Hi*CuAO2, *Hi*CuAO3, *Hi*CuAO4, and *Hi*CuAO5 were amplified using primer pairs P15/P16, P17/P18, P19/P20, and P21/P22, respectively (Table S1). Cloning into the binary vector pH7WG2D.1 (Karimi *et al*. 2007), transformation into *Agrobacterium tumefaciens* strain GV3101/pMP90, and transient expression in *N. benthamiana* leaves were performed as previously described (Zakaria *et al*. 2022). Infiltrated leaves were harvested 5 days after infiltration and pooled for protein extraction. All protein extraction and purification steps were performed as described in Zakaria *et al*. (2022).

### CRISPR/Cas9‐mediated genome editing in *H. indicum*

To design constructs for CRISPR/Cas9‐mediated genome editing, genomic DNA of *hicuao1*, *hicuao3*, and *hicuao5* was amplified using the primer pairs used for cDNA amplification (Table S1) to identify exon‐intron boundaries. Genomic DNA from leaves obtained from wild‐type plants was used as template, and PCR amplification was performed with the proofreading DNA polymerase, Phusion Hot Start II DNA polymerase (ThermoFisher Scientific, Waltham, MA, USA), according to the manufacturer's protocol, followed by cloning and sequencing. To identify protospacer sequences that could be used as short guide RNA (sgRNA) targets, the Geneious Prime software (v. 2020.1.2; Biomatters, Auckland, New Zealand) was used to predict possible target sites within the candidate genes. This software was also used to evaluate the on‐target activity of the different protospacer candidates using the algorithm of Doench *et al*. (2014), as well as to select the most specific guide RNA based on the scoring system of Hsu *et al*. (2013). Furthermore, we checked for off‐target interactions within and outside the selected gene using a custom off‐target database. This database was built from the identified genomic sequences of *hicuao1* to *5* and Illumina RNA‐seq data from *H. indicum* (Sievert *et al*. 2015), allowing a maximum of three mismatches. We only selected protospacers that showed the highest on‐target activity and specificity scores and a zero off‐target score. For each CuAO‐encoding sequence, two target sites were selected to increase the chance of obtaining a CRISPR/Cas9 effect or even a larger deletion of genomic sequence resulting from simultaneous cuts at both target sites. The constructs used for the genome editing approach were designed according to the protocol described by Schiml *et al*. (2016), which we used in a version recently adapted for genome editing in *S. officinale* (Zakaria *et al*. 2021). Briefly, complementary oligonucleotides covering the selected protospacer motifs were hybridized to produce sticky ends that allowed cloning into the BbsI‐linearized entry vectors pEn‐Chimera and pEn‐C1.1 (Schiml *et al*. 2016). The first sgRNA cassette from the resulting pEn‐Chimera construct was transferred by Gateway cloning into the destination vector pDe‐Cas9‐HYG (Zakaria *et al*. 2021), which contains the expression cassettes for Cas9 and for hygromycin resistance, as a plant selectable marker. This destination vector received the second sgRNA cassette containing the second protospacer from the pEn‐C1.1 construct (Schiml *et al*. 2016) by subcloning using MluI digestion. This resulted in pDe‐Cas9‐HYG constructs containing two sgRNA cassettes targeting two exons, namely construct A targeting exons 1 and 2 of the *hicuao1* gene, construct B targeting exons 1 and 3 of the *hicuao3* gene, and construct C targeting exons 2 and 4 of the *hicuao5* gene (Fig. 6). The sequences of the corresponding oligonucleotides are provided in Table S1.

### Induction of hairy roots in *H. indicum*

Hairy roots result from transformation by *A. rhizogenes*. They often grow faster than wild‐type roots, enabling a transgenic root system to be generated within weeks. These fine and branched roots (“hairy phenotype”) often exhibit the same biosynthetic capacity for secondary metabolite production as their mother plant roots, providing a robust and efficient tool for studying the function of target genes and pathways expressed in roots (Kim *et al*. 2002). *Agrobacterium*‐mediated transformation and generation of hairy roots in *H. indicum* was achieved according to a method described in Zakaria *et al*. (2021), with the following modification: transgenic HR lines that survived selection on hygromycin‐B‐containing medium were grown in the dark at 24°C on MS20 medium at a modified pH of 7.5.

### Analysis of HR lines for mutations within the *hicuao* genes and for polyamine and PA levels

Selected transgenic HR lines resulting from the CRISPR/Cas9‐mediated approach were analysed for mutations as described in Zakaria *et al*. (2021) using primer pairs overlapping the targeted exons of the *hicuao* genes (Table S1). The PCR products were purified using a PCR Clean‐up kit (Macherey‐Nagel) and sequenced either directly or after cloning into the pGEM‐T Easy vector (Eurofins Genomics, Ebersberg, Germany). Quantification of polyamines in HRs was performed as previously described in Zakaria *et al*. (2021), with the modification that 300 mg fresh weight hairy root material was used for extraction. PAs and CuAO‐catalysed reaction products were analysed after SPE purification according to a previously described method (Zakaria *et al*. 2022).

### Analysis of the genomic structure of *Hi*CuAOs

The complete genomic sequences of *hicuao1* to *5* of *H. indicum* were identified by amplification with the primer pairs listed in Table S1, followed by cloning into the pGEM T‐easy vector (Promega, Fitchburg, WI, USA) and sequencing (Eurofins Genomics, Hamburg, Germany). The resulting genomic sequences were compared to the identified cDNA to identify intron positions.

### Phylogenetic analysis

Transcript sequences of 38 CuAO‐like proteins from *H. indicum*, *S. officinale*, and other species that were available in public databases were used for phylogenetic analysis (Data S1). Sequence alignment and the phylogenetic tree construction were performed using the Geneious software package (Geneious Prime®, Biomatters, Auckland, New Zealand). Pairwise alignment options for building a distance matrix were Alignment type: MUSCLE alignment; Algorithm: PPP: The standard muscle algorithm. The tree was rooted using an algal CuAO from *Chara braunii*. The maximum likelihood phylogenetic tree was constructed using the BLOSUM62 substitution model for amino acid sequences. Bootstrap values ≥65% were inferred from 1000 replicates.

## RESULTS

### Root cultures of *H. indicum* are able to synthesize simple, rather than complex PAs

Earlier feeding experiments carried out by Frölich *et al*. (2007) revealed that roots of *H. indicum* are unable to incorporate [^14^C]Put into PAs. The recent finding that *S. officinale* uses specific leaves in addition to the roots to produce PAs (Kruse *et al*. 2017) inspired us to repeat and optimize such feeding experiments with a hairy root system. As it has been suggested that PA biosynthesis in *S. officinale* and *H. indicum* share a common ancestor (Reimann *et al*. 2004), we tested whether *H. indicum* is also able to synthesize PAs not only in aerial parts but also in roots.

**Fig. 1:**
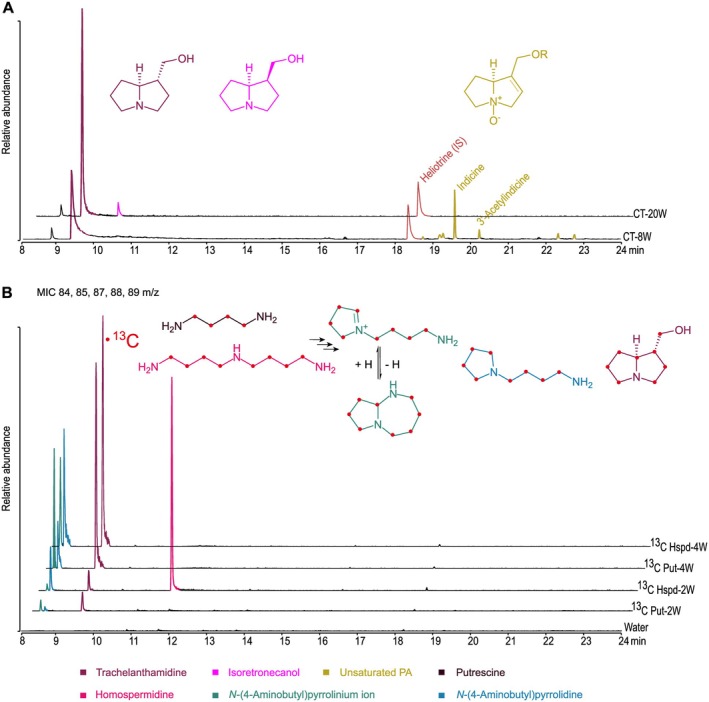
Analysis of hairy roots of *Heliotropium indicum*. (A) Gas chromatography‐total ion chromatograms (GC‐TICs) of hairy root extracts from transgenic control lines (CT) transformed with the empty binary vector pB2GW7.0. After 8 weeks, trachelanthamidine and traces of PAs were detected, the latter being most likely transferred from leaves to hairy roots during their early development. After 20 weeks, only trachelanthamidine was detectable at high levels, along with traces of its trans isomer, isoretronecanol. Heliotrine was used as internal standard (IS). (B) Multiple ion chromatograms (MICs) of hairy root extracts after feeding [^13^C]Put and [^13^C]Hspd for 2 and 4 weeks for the masses m/z 88 representing the base peak for fully labelled [^13^C]bicyclic intermediates, m/z 89 representing the base peak for fully labelled [^13^C]Hspd, and fully labelled [^13^C]monocyclic intermediates, m/z of 84 and/or 87 representing the base peak for half labelled [^13^C]bicyclic intermediates, and for the mass m/z of 85 and/or 88 representing the base peak for half labelled [^13^C]monocyclic intermediates.

Hairy roots (HRs) of *H. indicum* were generated and cultivated similar to the previously described experiments with *S. officinale* (Frölich *et al*. 2007; Zakaria *et al*. 2021, 2024), to allow longer incubations with sufficient root material. For infection of detached leaves of *H. indicum* with *A. rhizogenes*, the empty binary vector pB2GW7.0 (Karimi *et al*. 2007) was used. GC–MS analyses of the resulting hairy roots revealed that, after 8 weeks of cultivation, traces of complex PAs could be detected, together with high levels of trachelanthamidine, an unesterified pyrrolizidine backbone that we name here “simple PA” to distinguish it from the more complex PA esters (Fig. 1A). Of note, the pattern of complex PAs detected in these hairy roots was similar to that known from *H. indicum* leaves (Frölich *et al*. 2007; Zakaria *et al*. 2022). In hairy roots grown for 20 weeks, no complex PAs were detectable, not even in traces. Instead, the amount of trachelanthamidine increased significantly compared to 8‐week‐old hairy roots (Fig. 1A). In addition, isoretronecanol, the trans isomer of trachelanthamidine, was also detected in traces (Fig. 1A). Since the leaves of *H. indicum* used for infection and from which the hairy roots emerged are known to contain high levels of complex PAs (Frölich *et al*. 2007; Zakaria *et al*. 2022), we hypothesized that a possible transfer of complex PAs from the leaves to the roots occurred during the early stages of hairy root development. This hypothesis was supported by the observation that after a prolonged cultivation period of 20 weeks, these alkaloids were no longer detectable using GC–MS analyses. Obviously, the complex PAs have been diluted by the repeated subculturing procedure using only parts of the root material. Furthermore, these data indicate that the roots of *H. indicum* are able to synthesize simple unesterified PAs, *i.e*., trachelanthamidine and isoretronecanol, but not the complex PA esters, *i.e*., indicine and its 3′‐acetyl derivative, the main PAs reported in *H. indicum* aerial parts (Frölich *et al*. 2007; Zakaria *et al*. 2022).

### Roots of *H. indicum* have PA‐specific HSS and HSO

The observation that trachelanthamidine is produced *de novo* in the hairy roots suggests that roots of *H. indicum* are capable of catalysing the early steps of PA biosynthesis. To investigate this capacity in more detail, [^13^C]Put and [^13^C]Hspd were fed for 2 and 4 weeks to well‐developed hairy roots that had been cultured *in vitro* for several months and that were devoid of PAs. GC–MS analyses showed that [^13^C]Hspd was still detectable after 2 weeks, but not after 4 weeks (Fig. 1B). In all feeding experiments, no complex PAs were detectable (Fig. 1B). However, [^13^C]trachelanthamidine was well detectable in roots fed with [^13^C]Put and in roots fed with [^13^C]Hspd after 2 weeks and had even higher levels in roots grown for 4 weeks (Fig. 1B). In addition, the monocyclic *N*‐(4‐aminobutyl)pyrrolinium ion, as an intermediate of the HSO‐catalysed conversion of Hspd (Zakaria *et al*. 2022) and its reduced form, *N*‐(4‐aminobutyl)pyrrolidine (Zakaria *et al*. 2024), were detectable after both time intervals, with a higher accumulation of the reduced form after feeding with [^13^C]Hspd (Fig. 1B). Of note, fully labelled intermediates were detected not only after feeding with [^13^C]Hspd, but also after feeding with [^13^C]Put that provides only one of the two C4‐units of Hspd in the HSS‐catalysed reaction. This can be explained by the *in planta* conversion of [^13^C]Put to [^13^C]Spd and [^13^C]Hspd, of which only the aminobutyl moiety is labelled, by the action of Spd synthase (E.C. 2.5.1.16) and HSS, respectively. Subsequently, the [^13^C]aminobutyl moiety of these labelled substrates is transferred to the [^13^C]Put by HSS to form fully labelled [^13^C]Hspd (Ober *et al*. 2003). To exclude any artefact resulting from metabolic changes after hairy root induction, [^13^C]Put was fed to wild‐type *H. indicum* roots for 2 weeks. This feeding resulted in the same metabolic profile observed in the feeding experiments conducted with the hairy roots (Fig. S1).

**Fig. 2:**
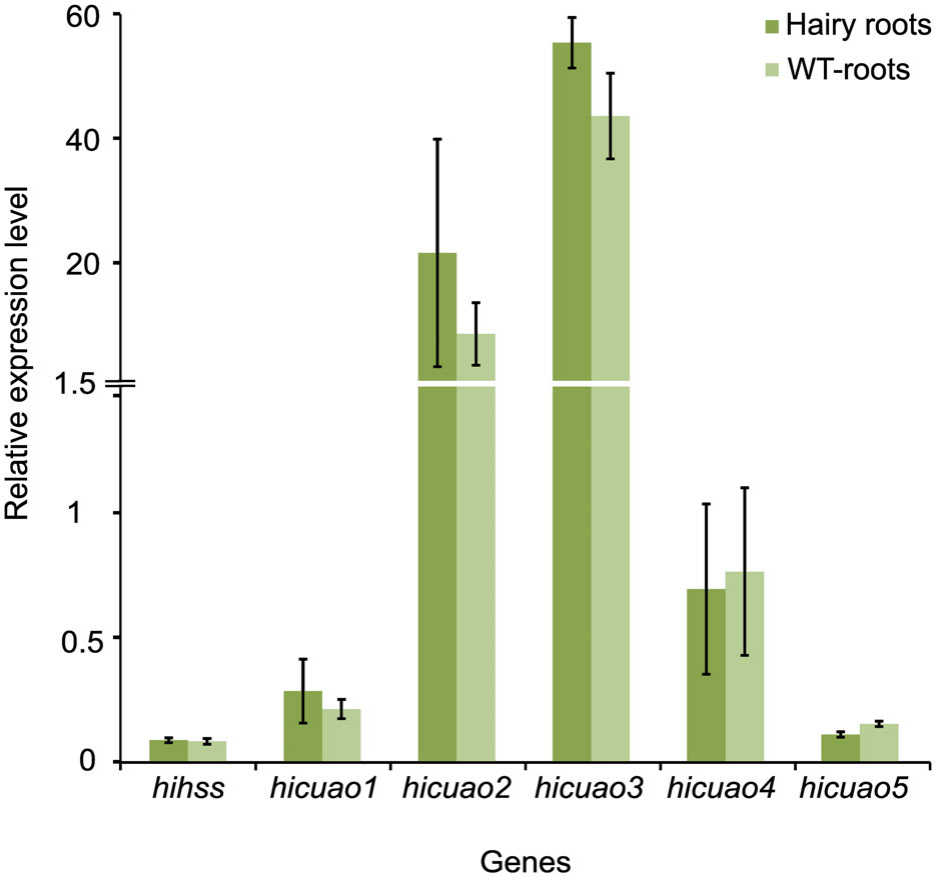
Relative expression level of the genes encoding homospermidine synthase (HSS) and copper‐containing amine oxidases (CuAOs) in hairy roots resulting from infection of detached leaves of *Heliotropium indicum* with *A. rhizogenes* harbouring the empty binary vector pB2GW7.0 and in wild‐type (WT) roots of *H. indicum* via RT‐qPCR analyses. Bar graphs represent mean ± SD of triplicate PCRs. Values were normalized to the ubiquitin gene as a reference gene.

The synthesis of trachelanthamidine involves, among other enzymes, an HSS and an HSO to convert Put and Spd via Hspd to the bicyclic necine base backbone. In particular, the oxidation of Hspd requires an enzymatic capacity that has recently been reported for the HSO from PA‐producing leaves of *H. indicum* (Zakaria *et al*. 2022), suggesting that such a specialized version of CuAO might also be expressed in the roots of *H. indicum*. Therefore, we performed RT‐qPCR analyses of the five recently identified genes encoding CuAOs of *H. indicum* (*Hi*CuAOs, Zakaria *et al*. 2022) and the gene encoding HSS of *H. indicum* (*Hi*HSS) to compare their expression patterns in hairy root cultures as well as in wild‐type roots of *H. indicum*. The gene expression profile was very similar between hairy root cultures and wild‐type roots (Fig. 2). The genes encoding *Hi*CuAO2 and *Hi*CuAO3 showed high transcript levels in the analysed roots in comparison to the genes encoding other CuAOs (Fig. 2). The same analysis also showed a very low transcript level of the gene encoding *Hi*HSS (Fig. 2).

### Phylogenetic analysis and gene structure study suggest a common origin of the PA‐producing CuAOs within the Boraginales

**Fig. 3:**
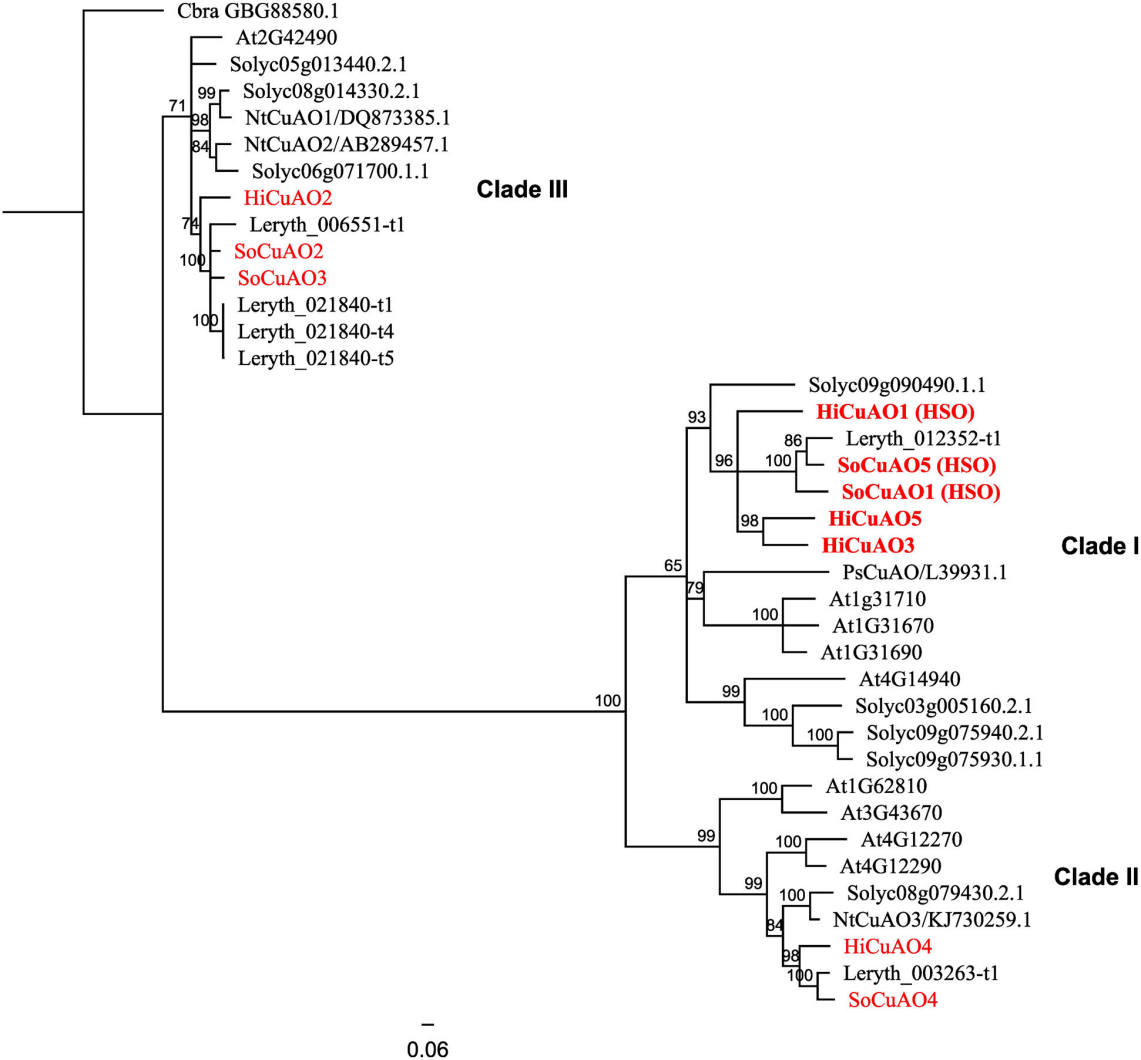
Phylogenetic analysis of 38 candidate copper‐containing amine oxidases (CuAOs). Phylogenetic tree of CuAO amino acid sequences from selected species together with CuAOs from *Heliotropium indicum* and *Symphytum officinale*. Sequence alignment and the phylogenetic tree were performed using the Geneious software package (Geneious Prime®, Biomatters, Auckland, New Zealand). Pairwise alignment options for building a distance matrix: Alignment type: MUSCLE alignment; Algorithm: PPP: The standard muscle algorithm. The tree was rooted using an algae CuAO from *Chara braunii*. The maximum likelihood phylogenetic tree was constructed using the BLOSUM62 substitution model for amino acid sequences. Bootstrap values ≥65% inferred from 1000 replicates are given on tree branches. Sequences highlighted in red are from *H. indicum* and *S. officinale*. Sequences in bold red are predicted to possess an *N*‐terminal signal peptide that directs them to the secretory pathway according to the SignalP‐5.0 server (Almagro Armenteros *et al*. 2019b) and the TargetP‐2.0 server (Almagro Armenteros *et al*. 2019a). All sequences used to calculate the tree are given in Data S1. Accession numbers are given for all sequences and in cases where biochemical information was available, the sequence name is given. At, *Arabidopsis thaliana*; Cbra, *Chara braunii*; Hi, *Heliotropium indicum*; Leryth, *Lithospermum erythrorhizon*; Nt, *Nicotiana tabacum*; Ps, *Pisum sativum*; So, *Symphytum officinale*; Solyc, *Solanum lycopersicum*.

For phylogenetic analysis, we used an alignment of the complete open reading frame (ORF) of CuAO sequences from various angiosperm species (listed in Data S1) and from the recently published sequences encoding CuAOs of comfrey (*S. officinale*; Zakaria *et al*. 2024), together with the sequences characterized in this study. Phylogenetic analysis shows that plant CuAOs form three well‐supported clades, named Clades I to III according to Tavladoraki *et al*. (2016). Both *Hi*CuAO3 and the 5 cluster with the previously characterized PA‐producing CuAOs, namely, *Hi*CuAO1 (*Hi*HSO, Zakaria *et al*. 2022), *So*CuAO1 and 5 (Zakaria *et al*. 2024), in Clade I. However, *Hi*CuAO2 and 4 are closely clustered with CuAOs most likely involved in primary metabolism, *i.e*., *So*CuAO2 and 3 in Clade III and *So*CuAO4 in Clade II, respectively (Fig. 3). The phylogeny supports a monophyletic group of all sequences that encode enzymes able to catalyse the formation of the bicyclic ring system. This suggests that the sequence Leryth_012352‐t1 from *Lithospermum erythrorhizon* also encodes an HSO. These observations imply that the common ancestor of these PA‐specific CuAOs in species belonging to the order Boraginales may already have been optimized for the necine base formation in PA biosynthesis.

**Fig. 4:**
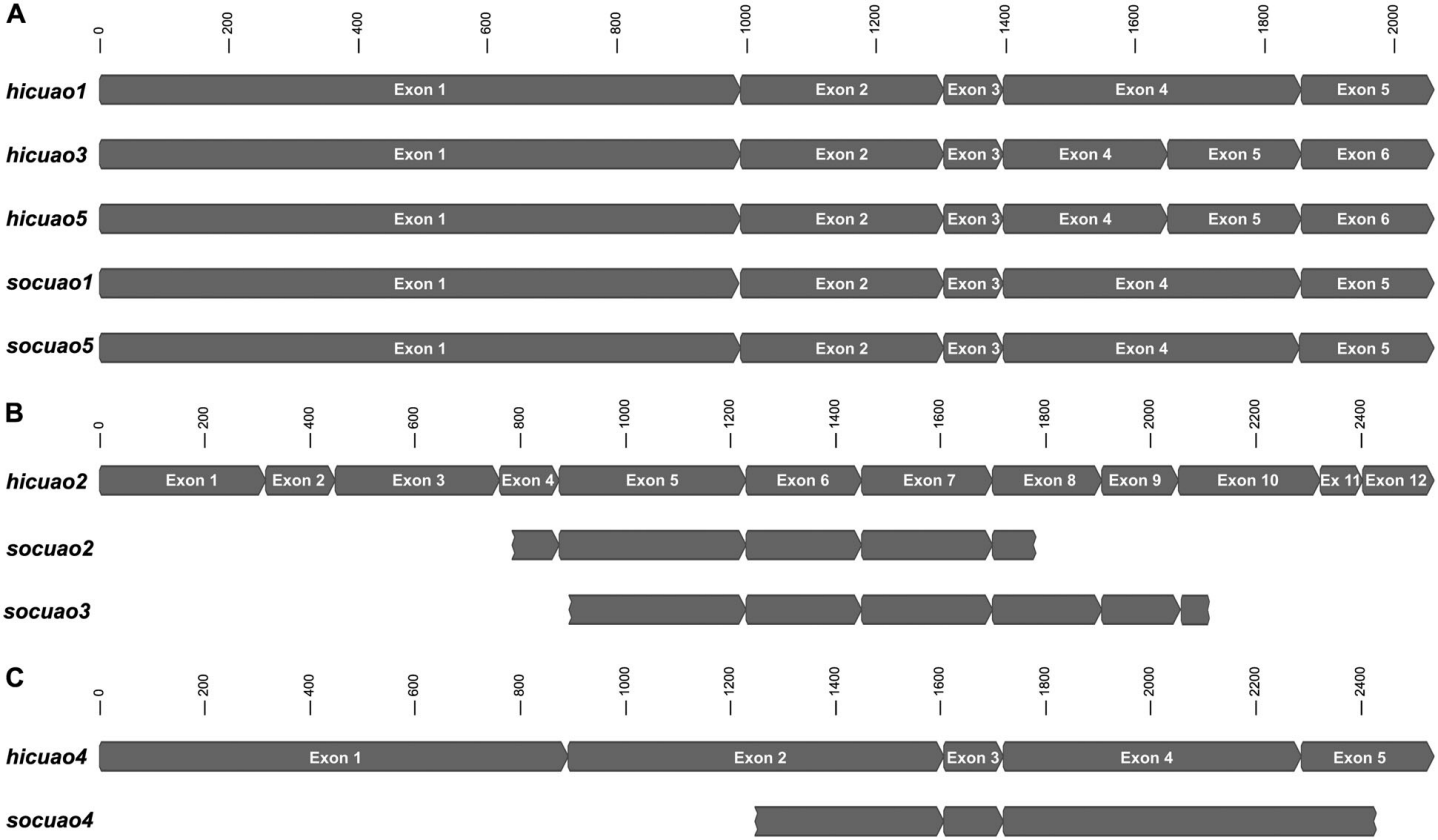
Schematic representation of an alignment of exon sequences of the copper‐containing amine oxidase (CuAO) genes of *Heliotropium indicum* and *Symphytum officinale*. These are grouped according to their similar exon patterns as follows: (A) *hicuao1*, *hicuao3*, *hicuao5*, *socuao1* and *socuao5*; (B) *hicuao2*, *socuao2* and *socuao3*; and (C) *hicuao4* and *socuao4*. Exon regions are represented by dark grey boxes. Boxes with flat left sides and pointed right ends represent fully identified exons, while boxes with ‘cracked’ sides represent partially identified exons. Exons are numbered only when the complete gene structure including the separating introns is identified, as in the case of *hicuao1* to *5* and *socuao1* and *socuao5*.

To gain further support for the relationship between the CuAOs under study, the number and position of introns within the ORF of the respective genes were analysed. The sequences obtained were compared with the described gene structure of the *cuaos* of *S. officinale* (*socuaos*; Zakaria *et al*. 2024). The genes *hicuao*1, 3, and 5, as well as *socuao*1 and 5 share a very similar gene structure with five exons separated by four introns for *hicuao*1, *socuao*1 and 5 and six exons separated by five introns for both *hicuao*3 and 5 at conserved positions (Fig. 4A). Since the *hicuao*3 and 5 sequences show the typical proto‐splice motif (A/C)AG||R at the insertion site of the additional intron 4, this suggests an intron insertion into exon 4 of the ancestor of *hicuao3* and *5* after separation from the *hicuao1* by gene duplication (Dibb & Newman 1989; Dibb 1991). High similarity in gene structure was also observed between *hicuao*2 and *socuao*2 and 3 (Fig. 4B), and between *hicuao*4 and *socuao*4 (Fig. 4C).

Of note, analyses using the SignalP‐5.0 server (Almagro Armenteros *et al*. 2019b) and the TargetP‐2.0 server (Almagro Armenteros *et al*. 2019a) predict that *Hi*CuAO1, 3, and 5, as well as *So*CuAO1 and 5 proteins, possess an *N*‐terminal signal peptide that directs them to the secretory pathway. No signal peptide was predicted for *Hi*CuAO2 and 4, or for *So*CuAO2, 3, and 4.

### Each of *Hi*CuAO1, 3, and 5 is sufficient for formation of the pyrrolizidine backbone

**Fig. 5:**
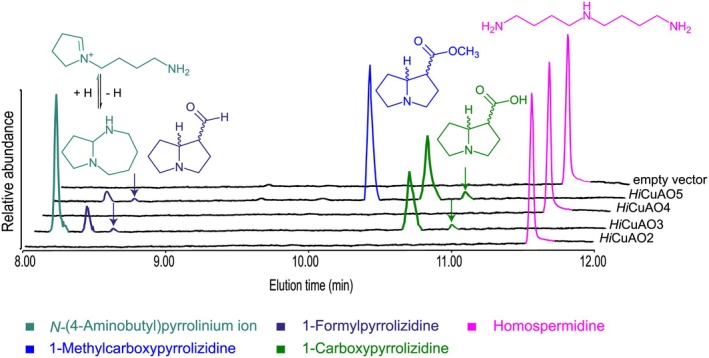
Identification of reaction products resulting from incubation of homospermidine with affinity‐purified *Hi*CuAO2, *Hi*CuAO3, *Hi*CuAO4 and desalted crude protein extracts of *Hi*CuAO5 and empty vector. Gas chromatography‐total ion chromatograms (GC‐TICs) of the purified reaction product mixture resulting from 48 h incubation with *Hi*CuAOs. The early eluting peak with a specific mass‐to‐charge ratio (*m/z*) of 140 represents the pyrrolinium ion in the deprotonated form (C_8_H_16_N_2_) due to the alkaline sample preparation, followed by a peak with *m/z* of 139 representing the bicyclic 1‐formylpyrrolizidine. Two later eluting peaks with (*m/z*) of 155 characteristic for bicyclic 1‐carboxypyrrolizidine and (*m/z*) of 169 consistent with the methyl ester of 1carboxypyrrolizidine resulting from methanolic sample preparation, are detectable. Arrowheads indicate minor isomers.

To identify the reaction products of identified and uncharacterized *Hi*CuAOs, *i.e*., *Hi*CuAO2 to 5, affinity‐purified proteins of *Hi*CuAO2 to 4 resulting from transient expression in *N. benthamiana* leaves were incubated with Hspd *in vitro*, followed by GC–MS analyses (Fig. 5). In the case of *Hi*CuAO5, which could not be purified after expression, desalted crude protein extracts of *N. benthamiana* leaves expressing *Hi*CuAO5 as well as of leaves infected with the empty vector control were used for incubation with Hspd under identical conditions. The purified proteins *Hi*CuAO2 to 4 and the desalted crude protein extract of *Hi*CuAO5 showed activity with Put as substrate in the CuAO‐specific colorimetric assay, as described by Zakaria *et al*. (2022, 2024). Only in incubations of *Hi*CuAO3 and *Hi*CuAO5 was the bicyclic pyrrolizidine identified, which was previously described as the product of *Hi*CuAO1 (*Hi*HSO; Zakaria *et al*. 2022). This bicyclic pyrrolizidine was detectable in the aldehyde form as 1‐formylpyrrolizidine (1‐fp) and in the acid form as 1‐carboxypyrrolizidine (1‐cp), the latter resulting from the non‐enzymatic oxidation of the aldehyde by the co‐produced hydrogen peroxide (Zakaria *et al*. 2022, 2024; Fig. 5). Of note, the two peaks for each of the bicyclic pyrrolizidines (1‐fp and 1‐cp) represent the two stereoisomers in an approximate ratio of about 10:1, which is consistent with the ratio of the two stereoisomers recently reported for the HSO from *H. indicum* (Zakaria *et al*. 2022) and the two HSOs from *S. officinale* (Zakaria *et al*. 2024). In addition, the monocyclic *N‐*(4‐aminobutyl)pyrrolinium ion resulting from a single oxidation of Hspd at only one of the two primary amino groups followed by a cyclization (Houen *et al*. 2005; Zakaria *et al*. 2022, 2024), and the methyl ester derivative of the carboxylic acid, most likely resulting from methanolic sample preparation, were also detected in these incubations (Fig. 5). In contrast, incubations with *Hi*CuAO2 and 4 showed only Hspd and none of the previously identified oxidation products (Fig. 5), indicating that Hspd is not accepted as a substrate. The *in vitro* enzyme activities show that the biochemical characteristics of both *Hi*CuAO3 and 5 are identical to the previously characterized *Hi*CuAO1 (*Hi*HSO; Zakaria *et al*. 2022), suggesting a possible involvement of any of these enzymes in PA biosynthesis in *H. indicum*.

### CRISPR/Cas9‐mediated inactivation of *Hi*CuAO1, 3, and 5 revealed that only *Hi*CuAO1 is involved in PA biosynthesis in *H. indicum* roots

**Fig. 6:**
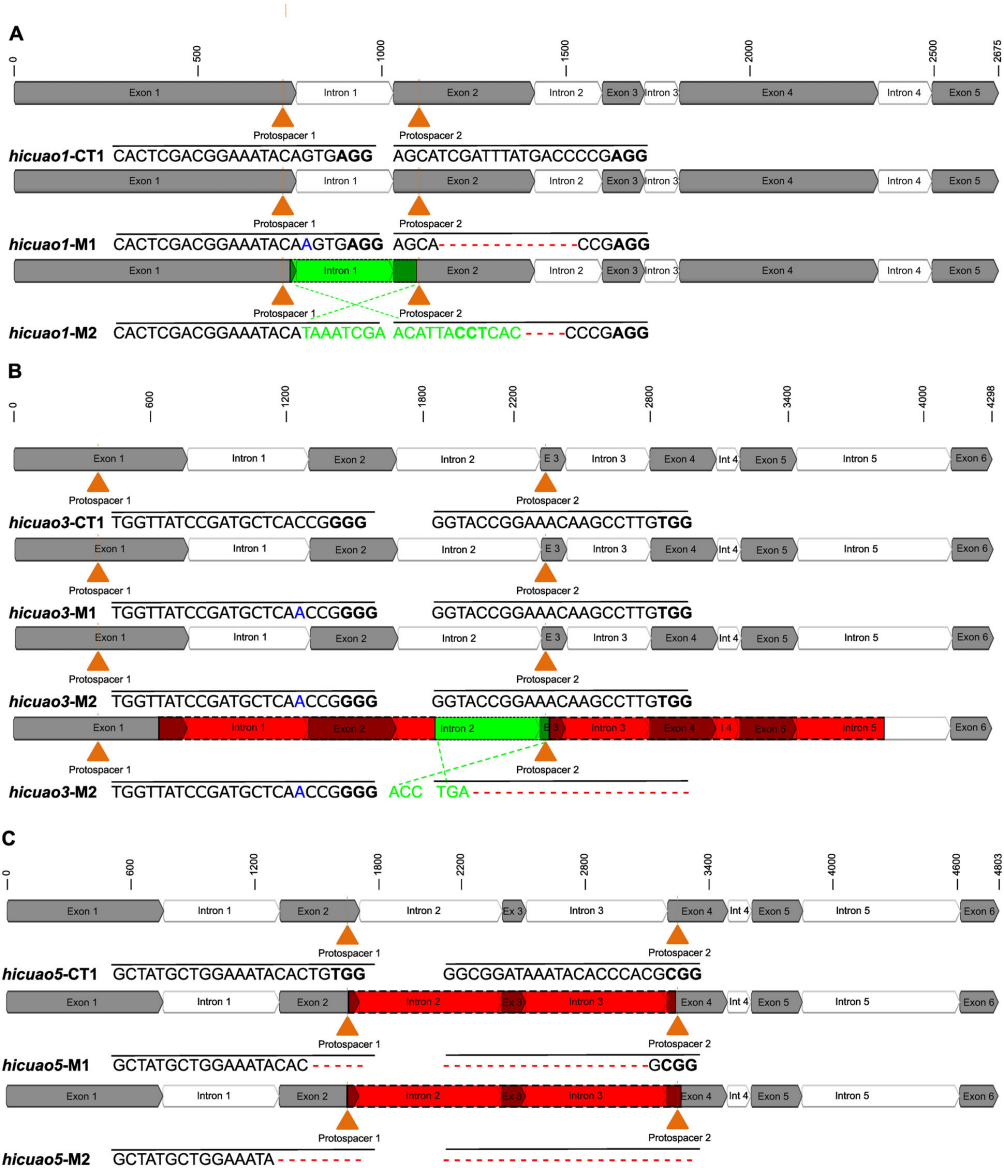
CRISPR/Cas9 editing approach and sequence analysis of induced mutations in transgenic HRs of Indian heliotrope resulting from different constructs targeting *hicuao1* (A), *hicuao3* (B), and *hicuao5* (C) genes. Exon regions are represented by dark grey boxes and intron regions are represented by white boxes. For all analysed lines, the 20 nt‐long protospacer sequences with the attached protospacer adjacent motif (PAM, NGG, bold) are shown in comparison to the sequence of the corresponding *hicuao* gene of the control line (*hicuao*‐CT1) resulting from transformation with the empty vector. The sequences of the *hicuao* genes of *hicuao*‐CT1 are identical to the unmodified *hicuao* genes of the wild type. The control line (CT) is always shown at the top and the mutant lines (M) are shown below it. The orange arrowhead indicates the position to which Cas9 is directed by the sgRNA in order to induce the double‐strand breaks. Dashes indicate deletions, blue nucleotides indicate insertions, red‐labelled parts indicate complete fragment deletions, and green‐labelled parts indicate flipped fragments as a result of simultaneous cutting at both target sites followed by reintegration of that fragment.

To test the relevance of *Hi*CuAO1, 3, and 5, which are all able to catalyse the formation of the bicyclic pyrrolizidine backbone *in vitro*, for PA biosynthesis *in planta*, we used CRISPR/Cas9 as a gene‐editing technique coupled to *A. rhizogenes* hairy root stable transformation, as described by Zakaria *et al*. (2022, 2024), to generate mutations that inactivate the corresponding genes. To knock out each gene, a construct containing two different short guide RNAs (sgRNAs), each with an N_20_ protospacer sequence targeting a different exon, was used. This resulted in construct A targeting exons 1 and 2 of the *hicuao1* gene, construct B targeting exons 1 and 3 of the *hicuao3* gene, and construct C targeting exons 2 and 4 of the *hicuao5* gene (Fig. 6).

Genomic DNA (gDNA) from HRs resulting from transformation with each construct was amplified and sequenced to reveal modifications within the sgRNA target sites (Fig. 6). Targeting *hicuao1*, two homozygous mutant HRs, designated *hicuao1*‐M1 and *hicuao1*‐M2, were obtained. In the *hicuao1*‐M1 line, a 1‐bp insertion mutation in exon 1 and a 13‐bp deletion mutation in exon 2 were observed. In the *hicuao1*‐M2 line, in addition to a 4‐bp deletion mutation in the target site in exon 2, the full‐length fragment between the two CRISPR target sites was still present, but in an inverted direction (Fig. 6A). Such flipping indicates that Cas9 has cut simultaneously at both target sites, resulting in excision of the entire fragment between the two cuts and its reintegration in reverse direction into the same genomic locus. Targeting *hicuao3*, two mutant lines, named *hicuao3*‐M1 and *hicuao3*‐M2, were obtained. While HR *hicuao3*‐M1 showed a homozygous mutation of 1‐bp insertion in exon 1, HR *hicuao3*‐M2 showed a biallelic mutation type. While the first allele showed a 1‐bp insertion mutation in exon 1, the second allele also showed an identical 1‐bp insertion mutation in exon 1 and additionally a 1172‐bp deletion mutation, together with a 503‐bp inverted DNA fragment between the two CRISPR target sites, and a 1475‐bp deletion mutation just after the second CRISPR site (Fig. 6B). Targeting *hicuao5*, two homozygous mutant lines with deletion of the complete DNA fragments between the two CRISPR/Cas9 target sites were obtained, with deletions of 1575‐bp and 1604‐bp in *hicuao5‐*M1 and *hicuao5*‐M2 HRs, respectively (Fig. 6C). Since all observed mutations resulted in either frameshift mutations or deletion of a large fragment of the gene, all these lines were considered as knockout mutants.

**Fig. 7:**
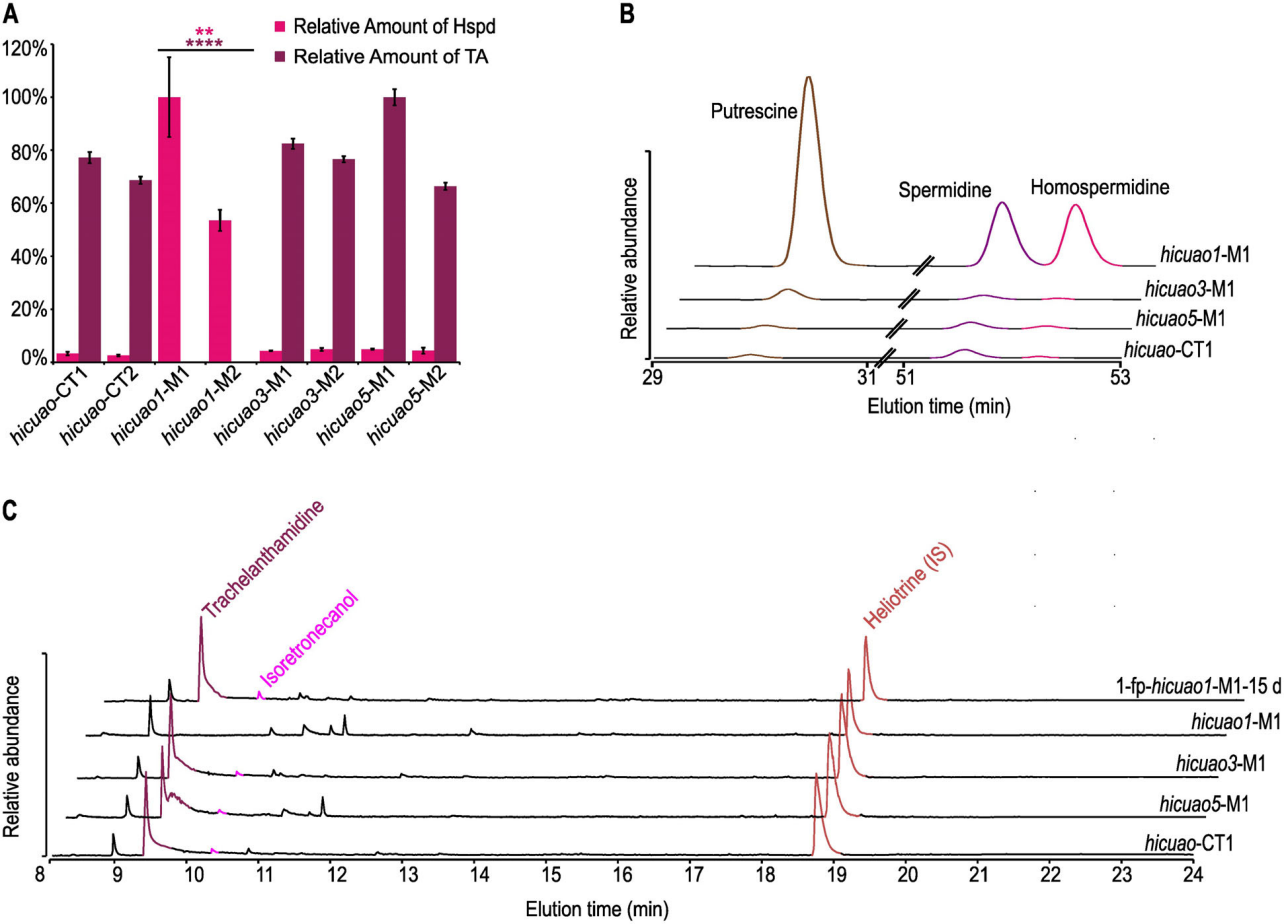
Chemotyping of transgenic hairy root lines. (A) Homospermidine (Hspd) and trachelanthamidine (TA) levels in hairy root lines resulting from transformation with the empty vector (control lines, *hicuao*‐CT) and the CRISPR/Cas9 constructs targeting the *hicuao1*, *hicuao3*, and *hicuao5* genes. Intensities have been calculated relative to the internal standards (IS) diaminoheptane and heliotrine and the amount of extracted biomass (fresh weight (FW) and dry weight (DW)), for quantification of Hspd and TA, respectively. 100% values correspond to 1765 nmol g^−1^ FW of Hspd and 30 μg g^−1^ DW of TA. Error bars represent SEM of three technical replicates of the same line (*n* = 3). Asterisks indicate statistical significance of differences in Hspd and TA levels between the TA‐free knockout lines (*hicuao1*‐M) compared to the control lines (**P < 0.01 and ****P < 0.0001). (B) Part of HPLC chromatogram showing the relative accumulation of the analysed polyamines (Hspd, Spd, and Put) in a mutant line (*hicuao1*‐M1) compared to a control line (*hicuao*‐CT1) and other mutant lines (*hicuao3*‐M1 and *hicuao5*‐M1). (C) Gas chromatography–total ion chromatograms (GC‐TICs) of purified extracts of HR lines showed in B in addition to the knockout HR line *hicuao1*‐M1 after feeding 1‐formylpyrrolizidine (1‐fp, the two stereoisomers in a ratio of 10:1) for 15 days.

To analyse the functional consequences of the CRISPR/Cas9 gene‐editing event observed in the generated knockout HRs on PA biosynthesis, Hspd was quantified by HPLC in all mutant lines resulting from constructs A, B, and C in comparison to the control lines resulting from transformation with the empty vector. Compared to the Hspd level of the various control lines (average = 51.42 nmol g^−1^ FW), only the *hicuao1* knockout lines showed a significantly increased level of Hspd (average = 1355 nmol g^−1^ FW, unpaired *t*‐test, **P = 0.0014; Fig. 7A). Of note, the levels of the other amines included in the polyamine analyses (*i.e*., Put, Spd) were also higher in these knockout lines than in the control lines (Fig. 7B). PA analyses of these lines showed that they are completely free of any type of PA, *i.e*., trachelanthamidine or more complex PA esters, whereas trachelanthamidine was still abundant in the control lines (Fig. 7A,C). On the other hand, both *hicuao3* and *hicuao5* knockout lines showed polyamine and trachelanthamidine levels similar to those observed in the control lines (Fig. 7A,B,C).

To confirm that the inability of the *hicuao1* knockout lines to produce trachelanthamidine is due to inactivation of the *hicuao1* gene, these lines were used for feeding experiments with the product of the *in vitro* incubation of Hspd with *Hi*CuAO1, *i.e*., 1‐formylpyrrolizidine (the two stereoisomers in a ratio of 10:1). After 15 days of feeding, 1‐formylpyrrolizidine was undetectable, while trachelanthamidine and its trans‐isomer isoretronecanol, were detectable, indicating the complete incorporation of the tracer into the bicyclic pyrrolizidine backbone (Fig. 7C). This suggests that the absence of PAs in the knockout lines is only due to the inactivation of the gene encoding *Hi*CuAO1 and not to any other off‐target effects of the CRISPR/Cas9 gene‐editing approach.

Taken together, these observations indicate that *Hi*CuAO1 is the only enzyme involved in Hspd oxidation as part of PA biosynthesis in *H. indicum* roots.

## DISCUSSION

Both *S. officinale* and *H. indicum* belong to the order Boraginales. PA biosynthesis shares a common origin in both species based on phylogenetic analyses of HSS‐encoding sequences (Reimann *et al*. 2004). However, tissue‐specific expression of PA biosynthesis is unique in these species. In *H. indicum*, complex PAs are constitutively synthesized in the lower leaf epidermis and the stem epidermis, as is the case for *S. officinale*, which produces complex PAs constitutively in the endodermis of a primary root. However, in the bundle sheath cells of specific leaves, PA biosynthesis in *S. officinale* depends on the flowering stage, as only leaves subtending the developing inflorescences produce PAs (Niemüller *et al*. 2012; Kruse *et al*. 2017). In this study, we have shown that roots of *H. indicum* are also able to synthesize PAs, but only as simple structures, *i.e*., the unesterified bicyclic necine base moiety as trachelanthamidine and isoretronecanol, most likely also constitutively, as the accumulation was observed over a long period of cultivation (Fig. 1A). Whether these simple PAs are transported to the aerial parts to be converted into more complex PA molecules remains to be further investigated. However, the fact that simple PAs are detectable only in hairy roots and detached roots after tracer feeding, but not in roots attached to the plant, supports the interpretation that these simple PAs are translocated and further diversified to complex PAs in the aerial parts. The apparent conflict with the data of Frölich *et al*. (2007), who described roots of *H. indicum* as not being a site of PA biosynthesis in tracer feeding experiments, is most likely related to the shorter incubation time of only 5 days after tracer feeding, and the focus of those analyses on detection of complex PAs like indicine and its 3′‐acetyl derivative. The observation that the gene encoding *Hi*HSS has very low transcript level (Fig. 2) is consistent with the data of Niemüller *et al*. (2012), who showed a very low, but unequivocal, expression of HSS in roots of *H. indicum*, and with the data of Frölich *et al*. (2007), who reported the ability of roots of *H. indicum* to synthesize low levels of Hspd. The low level of expression of the PA‐specific genes detected (Fig. 2) is also consistent with the slow biosynthetic conversion of the tracer observed in the feeding experiments conducted with *H. indicum* roots. Comparing the results of the feeding experiments conducted in the current study with the feeding experiments previously performed using leaves of *H. indicum* (Zakaria *et al*. 2022), *H. indicum* roots show a lower biosynthetic rate, as Hspd was incorporated only slowly into trachelanthamidine, being still detectable after 2 weeks of feeding (Fig. 1B). Furthermore, the monocyclic intermediates resulting from the first HSO‐catalysed oxidation of Hspd accumulate to levels never observed in the feeding experiments with *H. indicum* leaves (Fig. 1B). Considering that the same HSO enzyme is involved in both tissues in Hspd oxidation with the same kinetics, this variability in the biosynthetic conversion is most likely related to the significant difference in the expression profile of the PA‐specific genes (Fig. 2; Zakaria *et al*. 2022). This interpretation, that PA biosynthesis is dependent on the amount of transcript, is supported by previous observations that PA levels are reduced in *S. officinale* after silencing of HSS by RNAi (Kruse *et al*. 2017). A similar observation was made in experiments where HSS of *S. officinale* was edited by CRISPR/Cas9. When only one allele was inactivated, PA biosynthesis was still running, but at a significantly lower level than in the control lines (Zakaria *et al*. 2021).

The slow incorporation of the tracer allowed detailed analysis of the intermediates between Hspd and necine base formation. Previous tracer feeding studies on PA biosynthesis in leaves of *H. indicum* and hairy root cultures of *Cynoglossum officinale*, a close relative of *S. officinale*, using [^14^C]Put have reported the accumulation of an unidentified “Metabolite X” as putative pathway intermediate between Hspd and trachelanthamidine (Frölich *et al*. 2007). The same study reported characteristics of this “Metabolite X” similar to those of polyamines, such as its high polarity and the HPLC detectability of its benzoyl derivative (Frölich *et al*. 2007). We hypothesize that “Metabolite X” is one of the intermediates resulting from the HSO‐catalysed reaction. We exclude 1‐formylpyrrolizidine as being “Metabolite X” because it is not detectable due to efficient conversion after feeding and because it cannot be derivatized with benzoyl chloride. Since neither acyclic nor bicyclic intermediates preceding trachelanthamidine were detectable in our various feeding experiments, we propose that “Metabolite X” is either the detected pyrrolinium ion or its reduced form, *i.e*., the pyrrolidine (Fig. 1B). Both structures result from the oxidation of only one of the two primary amino groups of Hspd. The second free amino group would explain the polyamine behaviour and can be easily derivatized with benzoyl chloride. Of note, the pyrrolidine, as a reduced form, was only detectable in the feeding experiments with the roots (Fig. 1B), but not in our *in vitro* analyses of CuAOs with Hspd (Fig. 5). Therefore, the pyrrolidine could result from an *in planta* neutralization process to stabilize the highly reactive pyrrolinium ion after feeding unphysiologically high amounts of Hspd. However, the observation that the pyrrolidine arises from Hspd and is consumed again during longer feeding periods, as observed in the current and a previous study with leaves of *H. indicum* (Zakaria *et al*. 2022), suggests that it is also a precursor of PAs in these plants. This is in accordance with a feeding experiment conducted by Kelly & Robins (1988), who reported the incorporation of the pyrrolinium ion and the corresponding pyrrolidine into PAs. Since we were unable to detect the pyrrolidine in our *in vitro* analyses, it is tempting to suggest that the metabolic flux is different *in planta*, resulting in the transient accumulation of intermediates that are not detectable *in vitro*.

*Hi*CuAO1, 3, and 5 are capable of catalysing the double oxidative deamination and the cyclization of Hspd to form the pyrrolizidine skeleton *in vitro* (Fig. 5). This reaction is described as a characteristic feature of the PA‐specific CuAOs, *i.e*., *Hi*HSO (=*Hi*CuAO1), *So*CuAO1 and 5, which have been shown by gene editing to be involved in PA biosynthesis *in planta* (Zakaria *et al*. 2022, 2024). However, CRISPR‐Cas9 gene‐editing of the individual genes encoding *Hi*CuAO1, 3, and 5 has revealed that only *Hi*CuAO1 is involved in PA biosynthesis in *H. indicum* roots, while *Hi*CuAO3 and 5 are not. Of note, the *hicuao1* knockout HRs, which accumulate Hspd, also had higher levels of Put and Spd than the control lines and the *hicuao3* and *5* knockout HRs (Fig. 7B). This observation is in contrast to a previous study in which *socuao5* knockout HRs of comfrey accumulated Hspd but had similar levels of Put and Spd as the control lines (Zakaria *et al*. 2024). This suggests a different regulation of the polyamine pool in *H. indicum* roots. Furthermore, no compensation for the non‐functional *Hi*CuAO1 by either *Hi*CuAO3 or 5 was observed after CRISPR‐Cas9‐mediated inactivation, not even in traces, suggesting that *Hi*CuAO3 and *Hi*CuAO5 have no access to the substrate Hspd. A similar observation has been reported for HSS of comfrey. Knockout HRs of *hss* that still produced traces of Hspd through ubiquitous DHS were unable to incorporate this Hspd into PAs (Zakaria *et al*. 2021). This could be explained by a subcellular compartmentalization of PA biosynthesis that requires specific transport processes. Such phenomena are well known for the biosynthesis of certain classes of secondary metabolites (Dewey & Xie 2013; Beaudoin & Facchini 2014; Dobritzsch *et al*. 2016; Adebesin *et al*. 2017; Payne *et al*. 2017) and might also be relevant for PA biosynthesis. Furthermore, the *hi*cuao3 and 5 knockout HRs did not show any conspicuous phenotypic differences compared to the control lines, suggesting that these genes are at least not required for the development and growth of the HRs, and are most likely just redundant duplicates of the PA‐specific *Hi*CuAO1 that are still biochemically active (Krakauer & Nowak 1999). Also, in *S. officinale*, a gene duplication resulted in two paralogs of the PA‐specific CuAOs, but in this case they are subfunctionalized, as each is involved in only one of the two PA‐producing tissues (Zakaria *et al*. 2024). Phylogenetic analyses supporting a monophyletic group of all sequences encoding enzymes that can catalyse the formation of the bicyclic ring system suggest that Leryth_012352‐t, a sequence from the PA‐producing species *Lithospermum erythrorhizon* (Roeder & Rengel 1990), is most likely the ortholog to the PA‐specific CuAOs identified from *H. indicum* and *S. officinale* and also encodes an HSO. The function of the CuAOs of Clade I in species that do not produce PAs remains an open question (Fig. 3). However, for at least one sequence, *i.e*., the CuAO of pea seedling, Robins (1982) showed that also this enzyme is able to convert Hspd to the bicyclic pyrrolizidine backbone *in vitro* in a biomimetic experiment. Future studies will need to reveal in which metabolic processes these enzymes are involved.

## CONCLUSION

The CuAO phylogeny, together with the comparative gene structure analyses, suggest a common evolutionary origin of the PA‐producing CuAOs within the Boraginales, *i.e*., *Hi*CuAO1 with its paralogs *Hi*CuAO3 and 5 and *So*CuAO1 and 5, as previously established for HSS (Reimann *et al*. 2004). Although the identification of the second site of PA biosynthesis in *H. indicum* further supports the similarities between Indian heliotrope and comfrey plants, a different recruitment of these CuAOs at the two identified sites and a diverse organization of PA metabolism were observed. This adds a further aspect of biosynthetic complexity to the plant specialized metabolism.

## AUTHOR CONTRIBUTIONS

Conceptualization: MMZ; Formal analysis: MMZ; Investigation: MMZ, MS; Writing – original draft: MMZ; Writing – review and editing: MMZ, DO; Visualization: MMZ, MS; Supervision: MMZ, DO; Funding acquisition: DO, MMZ.

## Supporting information


**Fig. S1.** Analysis of wild‐type roots of *Heliotropium indicum* after feeding [^13^C]Put for 2 weeks. Multiple ion chromatograms (MICs) of wild‐type root extracts for the masses m/z 88 representing the base peak for fully labelled bicyclic intermediates, m/z 89 representing the base peak for fully labelled [^13^C]bicyclic intermediates, m/z 89 representing the base peak for fully labelled [^13^C]Hspd, and fully labelled [^13^C]monocyclic intermediates, m/z of 84 and/or 87 representing the base peak for half labelled [^13^C]bicyclic intermediates, and for the mass m/z of 85 and/or 88 representing the base peak for half labelled [^13^C]monocyclic intermediates.

**Fig. S2.** Images of infected leaves and selected HR lines of *Heliotropium indicum*.

**Table S1.** Primer sequences.

**Data S1.** Sequences used to calculate the phylogenetic tree of CuAOs.

## Data Availability

Sequence data from this article have been deposited in the GenBank data library under the following accession numbers: *Hi*CuAO1_genomic (PV067409), *Hi*CuAO2_genomic (PV067410), *Hi*CuAO3_genomic (PV067411), *Hi*CuAO4_genomic (PV067412) and *Hi*CuAO5_genomic (PV067413).
