# Supplementary figures and images for: Roots of Indian heliotrope (Heliotropium indicum) produce simple pyrrolizidine alkaloids using the same homospermidine oxidase involved in biosynthesis of complex pyrrolizidine alkaloids in aerial parts
Source: Plant Biol (Stuttg). 2025 Jul 18;27(7):1378–90. doi: 10.1111/plb.70077 (PMC12631511; doi:10.1111/plb.70077)

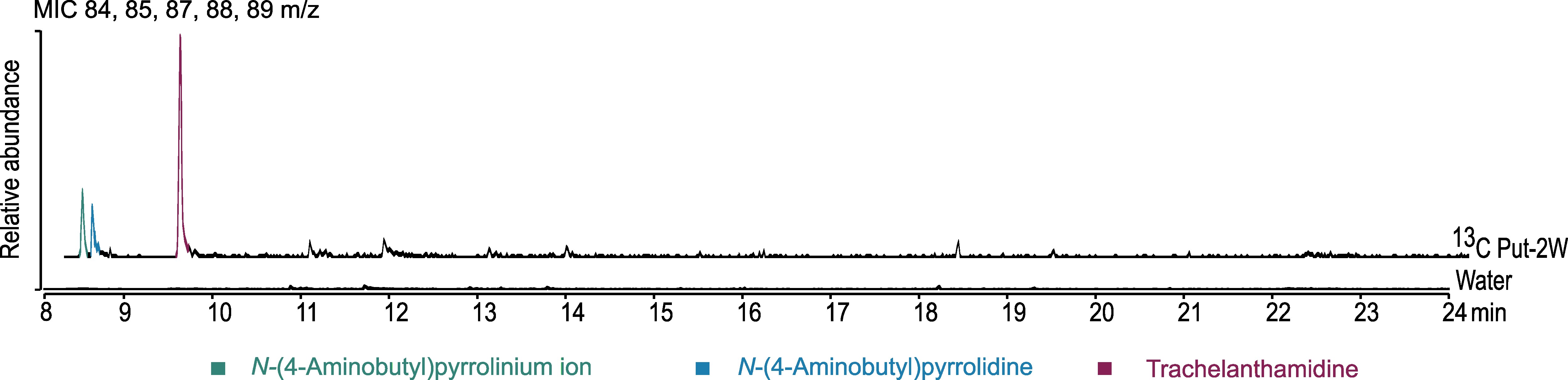

Supplement: Supplementary file 1 — Fig. S1. Analysis of wild‐type roots of Heliotropium indicum after feeding [13C]Put for 2 weeks. Multiple ion chromatograms (MICs) of wild‐type root extracts for the masses m/z 88 representing the base peak for fully labelled bicyclic intermediates, m/z 89 representing the base peak for fully labelled [13C]bicyclic intermediates, m/z 89 representing the base peak for fully labelled [13C]Hspd, and fully labelled [13C]monocyclic intermediates, m/z of 84 and/or 87 representing the base peak for half labelled [13C]bicyclic intermediates, and for the mass m/z of 85 and/or 88 representing the base peak for half labelled [13C]monocyclic intermediates. [file PLB-27-1378-s003.jpg]

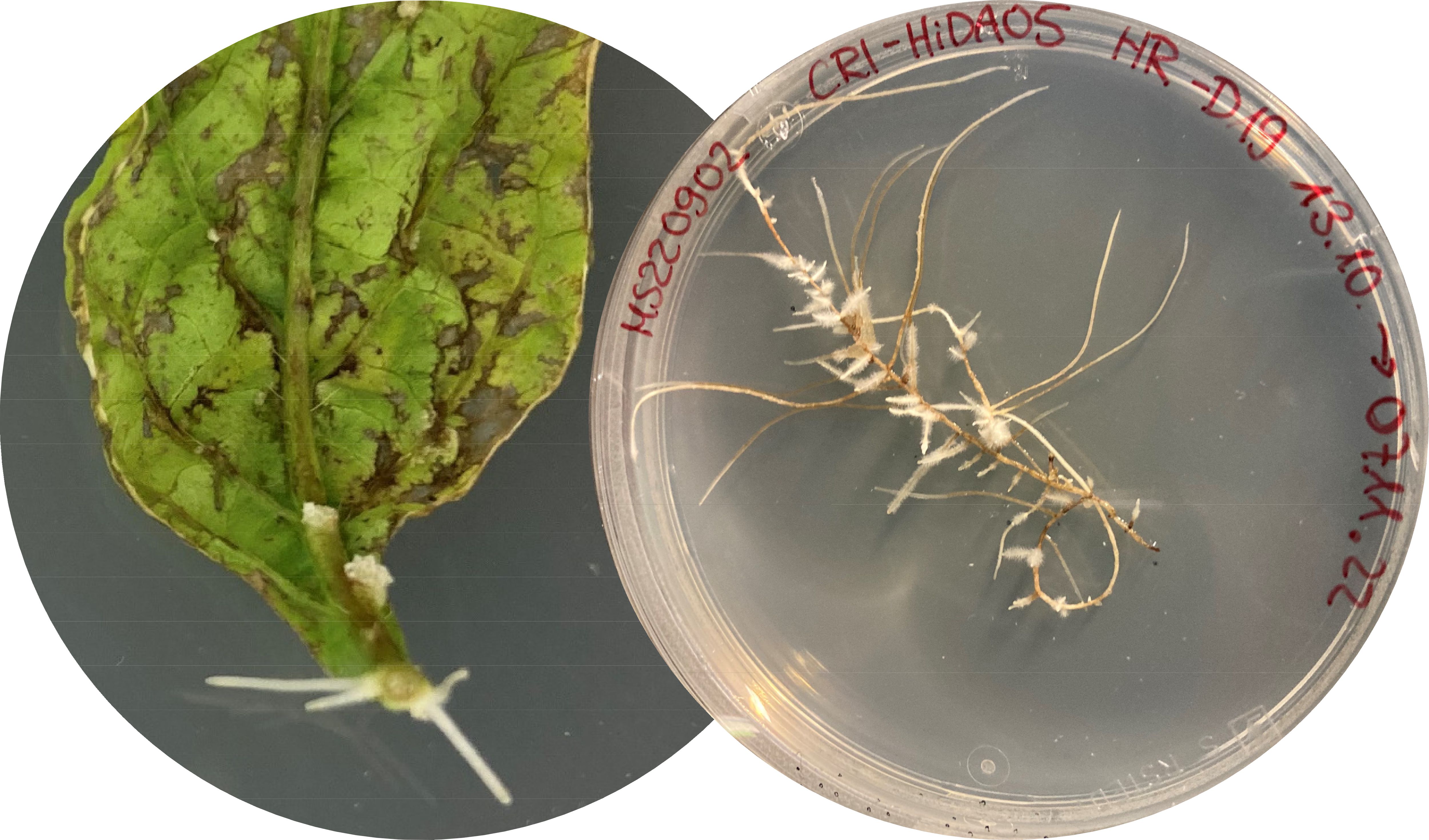

Supplement: Supplementary file 2 — Fig. S2. Images of infected leaves and selected HR lines of Heliotropium indicum. [file PLB-27-1378-s004.jpg]
