# Supplementary material for: Roots of Indian heliotrope (Heliotropium indicum) produce simple pyrrolizidine alkaloids using the same homospermidine oxidase involved in biosynthesis of complex pyrrolizidine alkaloids in aerial parts
Source: Plant Biol (Stuttg). 2025 Jul 18;27(7):1378–90. doi: 10.1111/plb.70077 (PMC12631511; doi:10.1111/plb.70077)
Supplement: Supplementary file 2 [file PLB-27-1378-s001.odt]

[Data S1](https://pmc.ncbi.nlm.nih.gov/articles/PMC520970/#tbl1)

Cbra GBG88580.1

MPRLGRRRREELAVAVMAIASRMAEDAFESLDSLLGNHTCRPLLLGPDNDMAGRALGEDDDDQTWGRRSLVWLFPTIYDICKLSPRTSPRWWASSMLKRLSESRDAVRQCCNDLVVNNMQIRRQVDRWRRAADDMRERFEHASHALDDVVQQRDNAIRRLESAEADLRVTRSQAETTWRFYDEVLDADDGVACGGIAGDKDLTTQFERIQPEGPSFRVDRYAVEWQKWSFRIGFTPKEGLVLHTVAYDDGDRGRRSIAHRLSFVEMVVPYGDPNGCDCLGFIKYFDAHFTNYVGGVETIENCVCLHEEDFGVLWKHQDWRTGWSEVRRSRRLSASFFCTLANYEYGFFWHFYQDGHIEAQVKLTGILSVGSLRDGESRPHGTVIAPGLYAPVHQHFFVARLDMAVDSKPGEGHNQVVEVNVRSDEPGPANPHENAFRAEETILRTEMQAKRDCNLLTGRHWIIRNTRTVNRTGRPTGYRLQPGSNCLPLALPGAKFLRRAAFLKHNLWVTQYRHHELFPGGDFPNQNPRIGEGLPTWVARNARIDNHNVVLWYVFGIIHVPRLEDWPVMPVEMIGFSLMPHGFFDCSPAIDVPPSQPAESTTPPSPSSVELHGKSDVIAAIRSKL

At2G42490

MASASKKTSACPHHGGSLPPPKLVSAAPDTVAVWSDADDQRASKVSLESVIRPVDSFPDNTAKKPANKGISVMPRTETKHPLDPLSAAEISVAVATVRAAGANPEVRDGMRFIEVASVEPDKQVVALADAYFFPPFQPSLLPRTKSGPVIPMKLPPRRARLVVYNQKSNETSVWIVALSEVHAVTRGGHHRGRVVSSQVIPDVQPPMDAAEYAECEAIVKDFPPFIEAMKRRGIEDMDLVMVDPWCVGYHSEADAPSRRLAKPLIYCRTDSDSPMENGYARPVEGIYVLVDMQNMVVIEFEDRKFVPLPPPDPLRNYTPGESRGGVDRSDVKPLQIIQPEGPSFRVRGYFVEWQKWNFRIGFTPREGLVIHSVAYVDGSRGRRPVAHRLSFVEMVVPYGDPNEPHYRKNAFDAGEDGLGKNAHSLKKGCDCLGSIKYFDAHFTNFTGGVETIENCVCLHEEDHGILWKHQDWRTGLAEVRRSRRLTVSFLCTVANYEYGFYWHFYQDGKIEAEVKLTGILSLGALQPGETRKYGTTIAPGLYAPVHQHFFIARMDMSVDCKPAEAFNQVVEVNVRVDERGENNVHNNAFYAEEKLLKSEAVAMRDCDPLSARHWIVRNTRTVNRTGQLTGYKLVPGSNCLPLARPEAKFLRRAAFLKHNLWVTRYAPDEKFPGGEFPNQNPRAGEGLATWVKQNRSLEESDVVLWYVFGITHVPRLEDWPVMPVEHIGFTLMPHGFFNCSPAVDVPPNPCELETKESEVKEVVAPKALQTGLLSKL

Solyc05g013440.2.1

MASAQEKTTVSAPSRNASAASETEWNAGGTVTDVSDLSSTRSASSAAPSTLTAVIDSIDRPSAQNQQPAVKGLPTLLRAQTHHPLDPLTAAEISVAVATVRAAGSTPEVRDSMRFVEVVLVEPDKSVVALADAYFFPPFQPSLLPRTKGGPVIPSKLPPRKAKLIVYNKKSNETSIWIVELSQVHAVTRGGHHRGKVISSKVVPDIQPPMDAVEYAECEAVVKDFPPFRDAMKKRGIDDMDLVMVDAWCAGYFSDADAPNRRLGKPLIFCRTESDCPMENGYARPVEGIHIVVDMQNMVVIEFEDRKVVPLPPADPLRNYTAGETRGGVDRSDVKPLLIVQPEGPSFRVNGHFVEWQKWNFRIGFTPREGLVIYSVAYIDGSRGRRPVAHRLSFIEMVVPYGDPNEPHYRKNAFDAGEDGLGKNAHSLKKGCDCLGYIKYFDAHFTNFTGGVETIENCVCLHEEDHGILWKHQDWRTGLAEVRRSRRLTVSFICTVANYEYGFYWHFYQDGKIEAEVKLTGILSLGALLPGEVRKYGTTIAPGLYAPVHQHFFVARMDMAVDCKSGESHNQVVEVNARVEPPGENNVHNNAFYAEERLLRTELEAMRDCNPLTARHWIIRNTRTVNRTGQLTGYKLVPGSNCLPLAGSEAKFLRRAAFLKHNLWVTPYSREEMFPGGEFPNQNPRVGEGLATWVQQNRSLEETQIVLWYIFGLIHVPRLEDWPVMPVEHIGFMLQPHGFFNCSPAVDVPPSMSDSDIKENGTVAKSCHNDGVMSKL

Solyc08g014330.2.1

MATNSKKVTKSFTNSCPSTSLLLRRETTAATAVVVDDGPQKWTVIPSVDDNKQKKTSSSAIASLASNTEPLPSNTSTKGIQIMLRAQTCHPLDPLSAAEISVAVATVRAAGETPEVRDGMRFIEVVLLEPDKSVVALADAYFFPPFQSSLMPRTKGGSLIPTKLPPRRARLIVYNKKTNETSIWIVELTEVHAAARGGHHRGKVISSNVVPDVQPPIDAQEYAECEAVVKNYPPFREAMKRRDIDDMDLVMVDPWCVGYHSEADAPNRRLAKPLVFCRSESDCPMENGYARPVEGIHVLVDVQNMQIIEFEDRKLVPLPPVDPLRNYTAGETRGGVDRSDVKPLHIIQPEGPSFRISGNYVEWQKWNFRIGFTPREGLVIHSVAYLDGSRGRRPIAHRLSFVEMVVPYGDPNDPHYRKNAFDAGEDGLGKNAHSLKRGCDCLGYIKYFDAHFTNFTGGVETTENCVCLHEEDHGMLWKHQDWRSGLAEVRRSRRLTVSFVCTVANYEYAFYWHFYQDGKIEAEVKLTGILSLGALQPGEYRKYGTTIAPGLYAPVHQHFFVARMNMAVDCKPGEAHNQVVEVNVKVEEPGKENVHNNAFYAEETLLRSELQAMRDCDPFSARHWIVRNTRTVNRTGQLTGYKLVPGPNCLPLAGPEAKFMRRAAFLKHNLWVTQYAPGEDFPGGEFPNQNPRAGEGLASWVKQDRPLEESDTVLWYIFGITHVPRLEDWPVMPVEHIGFMLQPHGFFNCSPAVDVPPPSGCDSETRDSDVTESTSVAKHTTTGLMAKL

NtCuAO1/DQ873385.1

MATTKQKVTAPSPSPSSSTASCCPSTSILRREATAAIAVVGDGLQNWTNIPSVDEKQKKTASSALASLPTTEPLSTNTSTKGIQIMTRAQTCHPLDPLSAAEISVAVATVRAAGETPEVRDGMRFIEVVLVEPDKSVVALADAYFFPPFQSSLMPRTKGGSQIPTKLPPRRARLIVYNKKTNETSIWIVELNEVHAAARGGHHRGKVIASNVVPDVQPPIDAQEYAECEAVVKSYPPFRDAMRRRGIDDLDLVMVDPWCVGYHSEADAPSRRLAKPLVFCRTESDCPMENGYARPVEGIYVLVDVQNMKIIEFEDRKLVPLPPVDPLRNYTAGETRGGVDRSDVKPLHIIQPEGPSFRISGNYVEWQKWNFRIGFTPREGLVIHSVAYLDGSRGRRPIAHRLSFVEMVVPYGDPNDPHYRKNAFDAGEDGLGKNAHSLKRGCDCLGYIKYFDAHFTNFTGGVETTENCVCLHEEDHGMLWKHQDWRTGLAEVRRSRRLTVSFVCTVANYEYAFYWHFYQDGKIEAEVKLTGILSLGALQPGEYRKYGTTILPGLYAPVHQHFFVARMNMAVDCKPGEAHNQVVEVNVKVEEPGKENVHNNAFYAEETLLRSELQAMRDCDPFSARHWIVRNTRTVNRTGQLTGYKLVPGPNCLPLAGPEAKFLRRAAFLKHNLWVTQYAPGEDFPGGEFPNQNPRVGEGLASWVKQDRPLEESDIVLWYIFGITHVPRLEDWPVMPVEHIGFVLQPHGYFNCSPAVDVPPPFACDSESRDSDVTETSVAKSTATSLLAKL

NtCuAO2/AB289457.1

MAATLHKVTPTTASASASIARRESAAASVLVDDQQKQTPALTSLLNSQPPSSNPSSKGKQIMPRAHTCHPLDPLSAAEISVAVATVRAAGETPEVRDGMRFIEVVLLEPDKSVVALADAYFFPPFQSSLMSRRKGGLPIPTKLPPRRARLIAYNKKTNETSIWIVELAEVHAAARGGHHKGKVISSNVVPDVQPPIDAQEYADCEAVVKNYPPFREAMKRRGIDDMDVVMVDPWCVGYHSEADAPSRRLAKPLVFCRTESDCPMENGYARPVEGIYALVDVQNMQVIEFEDRKLVPLPPADPLRNYTAGETRGGVDRSDVKPLQIIQPEGPSFRVNGNYVEWQKWNFRVGFTPREGLVIHSVAYLDGSRGRRPIAHRLSFVEMVVPYGDPNDPHYRKNAFDAGEDGLGKNAHSLKRGCDCLGYIKYFDANFANFTGGVETTENCVCLHEEDHGMLWKHQDWRTGLAEVRRSRRLTVSFICTVANYEYGFYWHLYQDGKIEAEVKLTGILSLGALPPGESRKYGTTIAPGLYAPVHQHFFVARMNMAVDCKPGEAHNQVVEVNVRVEEPGKENVHNNAFYAKETVLTSELQAMRDCDTLSARHWIVRNTRTSNRTGQLTGYKLVPGPSCLPLAGPEAKFLRRAAFLKHNLWVTQYAPGEDFPGGEFPNQNPRVGEGLASWVKQDRSLEESDVVLWYVFGITHVPRLEDWPVMPVEHIGFMLQPHGFFNCSPAVDVPPPRGCDLEIKDSDGSENGVAKPTPSSLMAKL

Solyc06g071700.1.1

MAETSQNVIPTPTNTTAAAAASFIPPTSVSVVRRENTAVSVSDSAVDSVVVDNQKEQTPATASSKGIQIMHRAHTCHPLDPLTAAEISVAVATVRAAGDTPEVRDGMRFVEVVLLEPDKTFVALADAYFYPPFQSSLMPRTKGGLLVPSKLPPRHARLIVYNKKTNETSVWIVRLTEVHAAVRGGQHRGKVISSKVIPDVQPPMDAQEYADCESVVKNYPPFIEAMKRRGIDDMDLVMVDPWCVGYHSEADAPSRRLAKPLIFCRTESYCPMENGYARPVEGIHVLVDVQIMQVIEFEDRKLVPLLPADPLRNYTAGETRGGVDRSDVKPLQIIQPEGPSFRVDGNYVQWQKWNFRVGFTPREGLVIHSVAYLDGSRGRRSIAHRLSFVEMVVPYGDPNDPHYRKNAFDAGEDGLGKNAHSLKRGCDCLGYIKYFDANFTNFTGGVETIKNCVCLHEEDHGMLWKHQDWRTGLAEVRRSRRLTVSFICTVANYEYGFYWHFYQDGKIEAEVKLTGILSLGALQPGESRKYGTTISPGLYAPVHQHFFVARMNMAVDCKPGEAHNQVVEVNLKVEEPGKENVHNNAFYAEETQLRSELQAMRDCDPLSARHWIVRNTRTSNRTGQLTGYKLVPGQNCLPLAGPEAKFLRRAAFLKHNLWVTQYAPGEDFPGGEFPNQNPRVGEGLASWVKEDRSLEESDIVLWYVFGITHVPRLEDWPVMPVEHIGFMLQPHGFFNCSPAVDVPPPRGCDSESKDSDASENGESKPTTTGLTAKL

HiCuAO2

MASTSEKATSAKCCDPPGDSSSVGVRREAASAAVVSREEWNEDAKNKSAAALASLINEPATKPTTKGIQYKAQSSHPLEPLSPAEISVAVSTVRAAGATPEIRDSMRFTEVVLLEPEKHVVALADAYFFPPFQPSLLPRTKTGDLVPIKLPPRRARLVVYNKKSNETSIWIVELTEVHATTRGGHHRGRVISSEVVPEVQPAVDAVEYAECEAIVKSHPPFIEAMRRRGIEDMDLVMVDSWCVGYYSEADSPCRRLAKPLVFCRTESDCPMENGYARPVEGCHVVVDVQNNVILEFEDRKFIPLPPVDPLRNYTRGATRGGVDRSDVKPLKIVQPEGPSFRVNGHYVEWQKWNFRIGFTPREGLVLHSLAYVDGDRGRRSIAHRLSFVEMVVPYGDPNEPHYRKNAFDAGEDGLGKNAHSLKKGCDCLGDVKYFDAHFTNYTGGVETLENCVCMHEEDHGILWKHQDWRTGLSEVRRSRRLTISFMCTVANYEYGFYWHLYQDGKIEAEVKLTGILSMGALQPGETRKYGTIIAPGLYAPVHQHFFVARMDMAIDCRPGEAHNQVVEVNVKIDEPGKDNVHNNAFYAEETLLKSELEAMRDCDPNSARHWIVRNTRIVNRTGQPTGYRLVPGSNCPPLARPEAMFLRRAAFLNHNLWVTKYARGEDFPGGEFPNQNPRVGEGLPSWVKQNRSLEEADIVLWYVFGVTHVPRLEDWPVMPVEHVGFMLQPHGFFNCCPGVDVPPNACEMDAKESSVKDNNGASLIAKL

Leryth_006551-t1

MASIAKKATPPGTDDVTIGRREAAATTEDWSGEESKPKKGSVISLIRPENATKSTTKGIQYMPRAQTRHPLDPLSAAEIVVAIATVRAAGATPEVRDSMRFVEVVLLEPDKQVVALADAYFFPPFQPSLLPRTRGGSMIPRDLPPRLARLVVYNKKSNETSVWIVQLSEVHAATRGGHHRGKVISSKVIPDVQPPMDAVEYAECEAVVKEYPPFVDAMKKRGIEDMDLVMVDAWLVLCVGYYGECDAPSRRLAKPLIFCRTESDCPMENGYARPVDGIHVLVDMQNMVLIEFEDRKLVPLPPADPLRNYTPGETRGGVDRSDVKPLQISQPEGPSFRVNGRYVEWQKWNFRIGFTPREGLVIHSVAYVDGSRGRRPIAHRLSFVEMVVPYGDPNEPHYRKNAFDAGEDGLGKNAHSLKKGCIYYLLDFTGGVETIENCVCMHEEDHGILWKHQDWRTGLAEVRRSRRLTVSFVCTVANYEYGFYWHFYQATCNFLNDGTNVAFAHPGMGGKPRDSADCLPAFSLWMDGKIEAEVKLTGILSLGALQPGEYRKYGTMIAPGLYAPVHQHFFVARMDMAVDCKPGEAHNQVVEVDVKVEGPGKDNIHNNAFYAEETLLKSELEAMRDCNPSSARHWIVRNTRTVNRTGQLTGYKLVPGSNCLPFSSPEAMFLRRAAFLKHNLWVTKYARGEDFPGGEFPNQNPRVGEGLASWVKQNRSLEETDIVLVSLLCYSTVLDKLLAVDQTKKGEYNFFFCLPHGFFNCSPAIDVPPSACELDIKENDAKDSAEMKQTPPNLIPKL

SoCuAO2

MASTTQKTTDQGDSTSARSPPDSKPAVISLISPEPATKPTTKGIQYMARAQTSHPLDPLSPAEIVVAIATVRAAGATPEVRDSMRFVEVVLLEPDKQIVALADAYFFPPFQPSLLPRTKGGSMIPRELPPRLARLVVYNQKSNVTSVWTVQLSQVHAATRGGHHRGKVISSKVVPDVQPPMDAVEYAECEAVVKEYPPFIDAMKKRGIEDMDLVMVDAWCVGYYSEADAPSRRLAKPLIFCRTESDCPMENGYARPVEGIHVVVDIQNMVLLEFEDRKLVPLPPADPLRNYTPGETRGGVDRSDVKPLKISQPEGPSFRVNGRYIEWQKWNFRIGFTPREGLVIHSVAYVDGSRGRRPIAHRLSFVEMVVPYGDPNEPHYRKNAFDAGEDGLGKNAHSLKKGCDCLGSIKYFDAHFTNFTGGVETIENCVCMHEEDHGILWKHQDWRTGLAEVRRSRRLTVSFMCTVANYEYGFFWHFYQDGKIEAEVKLTGILSLGALQPGEIRKYGTMIAPGLYAPVHQHFFVARMDMAVDCKPGEAHNQVVEVNVKVEGPGKGNVHNNAFYAEETLLRSELEAMRDCNPLSARHWIVRNTRTVNRSGQLTGYKLVPGSNCSPFSAPEAMFLRRAAFLKHDLWVTKYASGEDFPGGEFPNQNPRVGEGLASWVKQNRSLEETDIVLWYVFGITHVPRLEDWPVMPVEHIGFMLQPHGFFNCSPAVDVPPNVCELEVRENDVKDNAEMKQDPLSLIAKL

SoCuAO3

MASATKKATSPCCDQGNSSSAVVRREAAAGSLNVPPVESKEWAGESKSNKAAVISLIHTEPAAKSSTKGIQYMARAQTSHPLDPLSPAEIAVAIATVRAAGSTPEVRDSMRFVEVVLLEPDKQVVALADAYFFPPFQPSLLPRTKGGSMIPRELPPRLARLVVYNKKSNETSVWIVQLSQVHAATRGGHHRGKVISSKVIPDVQPPMDAVEYAECEAVVKEYPPFRDAMKKRGIEDMDLVMVDAWCVGYYNELDAPSRRLAKPLIFCRTESDRPMENGYARPVEGIHVVVDMQNMVLLEFEDRKLVPLPPADPLRNYTPGDTRGGVDRSDVKPLKISQPEGPSFRVNGRYVEWQKWNFRIGFTPREGLVIHSVAYVDGSRGRRPIAHRLSFVEMVVPYGDPNEPHYRKNAFDAGEDGLGKNSHSLKKGCDCLGSIKYFDAHFTNFTGGVETIENCVCLHEEDHGILWKHQDWRTGLAEVRRSRRLTVSFICTVANYEYGFFWHFYQDGKIEAEVKLTGILSLGALQPGESRKYGTMIAPGLYAPVHQHFFVARMDMAVDCKPGEAHNQVVEVNVKVEEPGSENIHNNAFFAEETLLRSELEAMRDCNPFSARHWIVRNTRTVNRSGQLTGYKLVPGSNCLPFSAPEAMFLRRAAFLKHNLWVTKYETGEDFPGGEFPNQNPRVGEGLASWVKQNRSLEETDVVLWYVFGLTHVPRLEDWPVMQWSI

Leryth_021840-t1

MASTSKKATAACCDQGDSSLGHRGTPAKSPAVPAASSASQEWNEDLKSTNAAVISLLRPETATKAIIKGIQYMARAQTSHPLDPLSPAEIAVAISTVRAAGATPEVRDSMRFVEVVLLEPDKQVVALADAYFFPPFQPSLLPRTKGGSIIPRELPPRLARLVVYNKKSNGTSVWIVQLSEVHAATRGGHHRGKVISSKVVPDVQPPMDAVEYAECEAVVKEYPRFIDAMKKRGIEDMDLVMVDAWCVGYYSEVDAPSRRLAKPLIFCRTESDCPMENGYARPVEGIHVVVDMQNMVLLEFEDRKLVPLPPADPLRNYTPGETRGGVDRSDVKLLKIVQPEGPSFRVNGRYVEWQKWNFRIGFTPREGLVIHSVAYVDGSRGRRPIAHRLSFVEMVVPYGDPNEPHYRKNAFDAGEDGLGKNAHSLKKGCDCLGYIKCFDAHFTNFTGGVETIENCVCMHEEDHGILWKHQDWRTGLAEVRRSRRLSVSFMCTVANYEYGFYWYFYQDGKIEAEVKLTGVLSLGALQPGEFRKYGTMIAPGLYAPVHQHFFVARMDMAVDCKAGEAHNQVVEVNVKVEGPGKENIHNNAFYAEETLLRSELEAMRDCNPRSSRHWIVRNTRTVNRSGQLTGYKLVPSSNCLPFSSPEAMFLRRAAFLKHNLWITHYASGEDFPGGEFPNQNPRVGEGLATWVKQNRSLEETDIVLWYVFGITHVPRLEDWPVMPVEHIGFMLQPHGFFNCSPAVDVPPNACELEIKENDVKDDAEMKPNPPSLIAKL

Leryth_021840-t4

MARAQTSHPLDPLSPAEIAVAISTVRAAGATPEVRDSMRFVEVVLLEPDKQVVALADAYFFPPFQPSLLPRTKGGSIIPRELPPRLARLVVYNKKSNGTSVWIVQLSEVHAATRGGHHRGKVISSKVVPDVQPPMDAVEYAECEAVVKEYPRFIDAMKKRGIEDMDLVMVDAWCVGYYSEVDAPSRRLAKPLIFCRTESDCPMENGYARPVEGIHVVVDMQNMVLLEFEDRKLVPLPPADPLRNYTPGETRGGVDRSDVKLLKIVQPEGPSFRVNGRYVEWQKWNFRIGFTPREGLVIHSVAYVDGSRGRRPIAHRLSFVEMVVPYGDPNEPHYRKNAFDAGEDGLGKNAHSLKKGCDCLGYIKCFDAHFTNFTGGVETIENCVCMHEEDHGILWKHQDWRTGLAEVRRSRRLSVSFMCTVANYEYGFYWYFYQDGKIEAEVKLTGVLSLGALQPGEFRKYGTMIAPGLYAPVHQHFFVARMDMAVDCKAGEAHNQVVEVNVKVEGPGKENIHNNAFYAEETLLRSELEAMRDCNPRSSRHWIVRNTRTVNRSGQLTGYKLVPSSNCLPFSSPEAMFLRRAAFLKHNLWITHYASGEDFPGGEFPNQNPRVGEGLATWVKQNRSLEETDIVLWYVFGITHVPRLEDWPVMPVEHIGFMLQPHGFFNCSPAVDVPPNACELEIKENDVKDDAEMKPNPPSLIAKL

Leryth_021840-t5

MARAQTSHPLDPLSPAEIAVAISTVRAAGATPEVRDSMRFVEVVLLEPDKQVVALADAYFFPPFQPSLLPRTKGGSIIPRELPPRLARLVVYNKKSNGTSVWIVQLSEVHAATRGGHHRGKVISSKVVPDVQPPMDAVEYAECEAVVKEYPRFIDAMKKRGIEDMDLVMVDAWCVGYYSEVDAPSRRLAKPLIFCRTESDCPMENGYARPVEGIHVVVDMQNMVLLEFEDRKLVPLPPADPLRNYTPGETRGGVDRSDVKLLKIVQPEGPSFRVNGRYVEWQKWNFRIGFTPREGLVIHSVAYVDGSRGRRPIAHRLSFVEMVVPYGDPNEPHYRKNAFDAGEDGLGKNAHSLKKGCDCLGYIKCFDAHFTNFTGGVETIENCVCMHEEDHGILWKHQDWRTGLAEVRRSRRLSVSFMCTVANYEYGFYWYFYQDGKIEAEVKLTGVLSLGALQPGEFRKYGTMIAPGLYAPVHQHFFVARMDMAVDCKAGEAHNQVVEVNVKVEGPGKENIHNNAFYAEETLLRSELEAMRDCNPRSSRHWIVRNTRTVNRSGQLTGYKLVPSSNCLPFSSPEAMFLRRAAFLKHNLWITHYASGEDFPGGEFPNQNPRVGEGLATWVKQNRSLEETDIVLWLV

Solyc09g090490.1.1

MLRFIASQLTMPLFFKISIFFFFVSTSLSSSPLEYNHPLESLTLFELTQVTNIVKNSYFPLNVTFHYVGLNEPNKQLVLSWLSSTHHPSLPREAIVITRIDQKTHEIIVDLSSVSILSDKIYNGHGYPMLTLQEQTAANELPLSYPPFISSIKKRRLKLKEVACQGYPVGWYGEEKKSTNRMVKVICYYLDGTVNLYMRPIEGITIIVLLDQMTIASYIDRLIVPVPKANGTDYMGSKRRRSSHESNSIKIVQPDGPSFTLDDGNTVRWVDWKFHVAYDMRAGIIISLASMFDDDKGEFRSVMYRGFASEVFVPYMDLTEEWYYRTYFNAGEFGFGLCAVELEPSKDCPENAKFIDGYFISQDGTPGKMPNVICIFERYAGDIMWRHTEDAIPGEKVGLTGLLEVRGSIYTHSDQIKEEVYGTLIAENTLGAYHDHFFTYHLDLDVDGHENSFVKNNLKTRRVIHKSSPRKSYWTVVSETAETESDAKIQLGSSRGAFEMVVVNPNKKTEIGNEIGYCLIPGGSATSPLLSDNDYPQARGGFTKYNVWVTPYNKSEKWAGGLYTDQSHGDDTLAVWSLRDRKIKNKDIILWYTFGVHHVPKQEDFPIMPTLSTGFELKPTNFFQHNPVL

HiCuAO1 (HSO)

MNSPLKFLLFFLFNYLSIASSNDQYNPLDPLTPSELTQTRDIVKGFINSTKNVTFHYVGLDEPDKQVVKSWLSSRNNMQTPPRKAFAIIRHDGKTHELVVDLATSSVISNNIYTGHGYPTLNFEEQELANQLATTYPPLVASMAKRGLKIEEVVCLTFTVGWFGEEKSNRVIKVQCYYLDGTVNFYMRPIEGITVTVDLDKMKVVGFRDRAIVPVPKAEGTEYIESKQVNPFQSNLKEKLVQSSEPSFTLDGNTVRWGNWQFHLKFDMRAGSVVSLASIYDPEEGGYRSVLYRGYVSEMFVPYMDLTEEWYYRVFFDSGEYGSGLCTGSLIPERDCPAGAVYMDGHLVGQDGTPRTIPKVFCLFERNAGDILWRHTEIGIPGKLYTEVRPETSLVARTVLTLSNYDYVIDYEFKKAGTIKVTAGLTGMIEVRAAEYTHKDQIKEEVYGRLVAEKTLGANHDHFLNYYLDLDIDGDANSFVKTKMQTKRVVGNQSPRKSYWIATSETVKTESNARIRLGAGDSELSFVNPNKKTKLGNEVGYRLIPGNVVGPLLTDDDYPQLRGAFSKYNVWVTPYNKSEKYAGGLYVDQSRGEDTLAVWSRRNRSIENRDIVLWYTLGFHHVPHQEDWPIMPTLTGGFELRPADFFEYNPVLYAQSTSEVE

Leryth_012352-t1

MSPSSSFLFFFLTISLASSQGFNHPFDPLAPLEITQVQTIVTSYISSSSSFTTNQKVHFHYVGLDEPDKPSINSWLSSPSLSSNLPPRQAFVIIRVGQQTHELKVDLSNNSVVSNMLHNGNGFPTITSEELEIANALPFEYPPFIASMDKRGLNVEEVVCESCTVGWFGEKEKSNRIVKVVCYYLNGTVNLFMRPIEGIIVVVDLDQMKVTGYNDRAIIPIPKAQGTDYRQVKETDGEGERRLFRSSLEEEIIMQQDDPNFSIEGHTVRWENWEFHLAFDMRAGPIISLASIFDPEQDTYRRVLYRGFLSEIFVPYMDLTLEWYFRVFFDAGEYGGGLCTVPLQPLTDCPRDAKFMDGYFTNQDGSFGKTPNVFCVFERDAGNVLWRHTETGIPNRVITEVRKEKTLVLRTVLTLSNYDYIVDWEFKKTGVITVTVGATGLLEVRGTEYTHADQIKEEAYGTLLAENSLGANHDHFLSFYLDLDIDGEANSFVKTKLETKRVTGNWSERKSYWTTVSEAAKTESDARVRVGSGDTQLVFVNPNKKTKIGNNIGYRLVPGSNVGQLLTDDDYPQIRGDFSKYNVWVTPFNKSERYAGGLYVDQSHGEDTLAEWSKRDREIENKDIVLWYTMGFHHVPCQEDFPLMPTLNGVFELRPYNFFEQNPVLNAKN

SoCuAO5 (HSO)

MPPSTHQAFLFFFFFLNIFLTSSSHPFDPLGQPELTQVQTIVTSFISSSSSNSSNQKIHFHYVGLDEPDKPAINSWLSSSTSSSNLPPRQAFVIIRVGKQTHELKVDLSNNSVVSNKLYNGNGFPTITSEELEIANALPLTYPPFIAAMEKRGLKMEEIVCESCTVGWFGEKETSNRIVKIVCYYMNGTVNIFMRPIEGIIVVVDLDQMKVTGLSDRAIIPVPKAEGTDYRQQNEAEGEGRFRSSLEEKVVMQQDEPNFSIDGHTVRWENWEFHLAFDMRAGPIISLASIFDPEKETYRRVLYRGFLSEIFVPYMDLTAEWYFRVFFDAGEYGGGLCTVPLQPLTDCPPDAKFMDGYFPNQDGSPAKTPNVFCVFERDAGDILWRHTETGIPNKLITEVRKEKTLVVRTVLTLSNYDYIVDWEFKKTGVITVTVGATGLLEVRGTEYTHTEQIKEEAYGKLLAENSVGANHDHFLSFYLDLDIDGESNSLVKTKLETKRVDGNWSERKSYWTTVSESAKTESEARLRVGSGETQLVFVNPNKKTKIGNNIGYRLVPGANVGQLLSDDDYPQIRGDFSKYNVWVTPFNKSERWAGGLYVDQSHGEDTLATWTNRDREIENKDIVLWYTLGFHHVPCQEDFPVMSTLNGGFELRPYNFFEQNPVLHAKN

SoCuAO1 (HSO)

MSPSTLKLSLLFFFFFFFFHIALLSHAFSHPLDPLAPPEITTVQTIVTSYISSSSTNEKVHFHYVGLDEPDKPTINSWLSSSTTSSSNLPPRQAFVIIRIGKQTRELEVDISNHSVVSNKLYNGNGSPTITSEEIDKSNDLPFKYPPFIASIEKRGLKLEEVVCGSCTIGWFGEKETSNRIVNVNCYYMDGTVNIYMRPIGGVVVMVDLDQMKVTGLRDRAIIPVPKAEGTDYRQEKEAAEEGEGRFRSSLEEKVTMQQDHPNFLLDGHLVRWENWEFHLAFDMRAGPIISLASIFDPEQDSYRRVLYKGFLSEMFVPYMDLTYEWYSRSFFDSGEYGGGLCTVPLQPLTDCPANAMFMDGYFPNQDGLPVKTPNVFCVFERDGQDILWRHTETGIPKRLITEVRKEKTLVVRTVLTLSNYDYIIDWEFKTTGVIKITVGATGLLEMRGTEYTHVDQIKEDVYGTLLAENSIGANHDHFLSFYLDLDIDGEANSFVKTKLETKRVNGNWSKRKSYWTPVSQTANTESEARIRVGSGDTQLVFINPNKKTKIGNNIGYRLYPGSNVGHLMSDDDYPQIRGDFSKYNVWVTPFNKSEIWAGGLYADRSHGDDTLAVWTERDREIKDKDIVLWYTLGFHHVPCQEDFPVMPTLNGVFELRPYNFFEHNPVLNAKNEL

HiCuAO5

MASSLNLLFPLLLISIFSSISIASYHPLDPLSPSELTQVHTILKKSITTRRNMTFHYVGLDEPDKPFVQSWLSNNNIKNIPPRKAFVIVRYDGKTHEIVVDLSTNSISSNKVYKGSGYPLLTPEEQTVANELPFKYPPFLASMKKRGLSLKEVVCVSMSIGWFGEGKTSRVVKVVGYYMNGTVNLYMRPIEGITITVDLDQMKIVEYLDREIVTIPKAEGTEYRESKQDGPLGSTISMKMNGQKINVSGHTVRWDNWEFHVSFDMRGGAMLSLASFYDPDKDERRQVIYQGHVAELFVPYMDLTEDWYYRTFFDSGEFGAGLCTVPLEPLRDCPENAVYLDGHFVGLDGTPAKIPNAMCLFERYAGNTLWRHTETAIAGKLLTEAIPETTLVVRSVFTLSNYDYIVDWEFKRTGTIKITTGLSGMLEVRADKYTHADQIKEEVYGAIIAENTLGTNHDHFLSFYLDLDIDGQANSFVKNKLTTKRVNGNGSKRKSYWTVVSETVKTESEAKLKLGSGNTELLVVNPNKKTNVGNSVGYRLVPESVVGPLLSEDDYPQIRAAFSNYNVWVTPYNKSEKYASGIYVDQGHGDDTLAVWTLRDREIENKDIVVWYTLGFHHAPHQEDFPIMPTLTGGFELRPTNFFDHNPLLNSKPSKKVNLT

HiCuAO3

MASTRNLFFPFLFISIFPLLSIASYSPLDPLTPSELNQVHAIVKNSVNTNDNLSFHYVGLDEPDKSAVQSWLLNGDASRIPSRKASVVVRYDGRTHELMVDLSTNSILSNELYNGYGYPMLTGEEQTAANELPMRYQPYVAAIKKRGLKLEEVVCLTMTIGWYGENETSRLVKVNSYYMDGTMNLYMRPIEGITVTVDLDQMKIVGFRDRLIVPVPKAKGTEYRLSQLPLRSNLEVNLRQPNEQRIDIDGNIVRWANWEFHLSFDMRAGAMLSLASYYDPEKEERRQVFYQAHVSELFVPYMDMTEEWYYRTFFDSGEFGAGLCTSSLVPLRDCPENAVYRDGYFVWEDGSLGTTPNAICIFERNAGDVLWRHTETALEGGMPTEAVPETSLVVRTVLTLSNYDYIVDWEFKLPGTIKVTTGLTGMLEVRGTEYTHADQIHEEVYGTMLAENTLGAFHDHFLNFYVDLDIDGEANSFVKNKLTTKRVKGNSSKRKSYWTVVSETVKTESDARIRLGTGESELLIVNTNKKTKVGNPVGYRLVPGSNVGPLLSDDDYPQIRGAFSKYNVWVTPYNKTERFAGGKYVDQSHGENTLAVWTLRNREIENKDIVLWYTLGFHHVPHQEDFPIMPTLTGGFELRPASFFEHNPLLNAKSTSEAK

PsCuAO/L39931.1

MASTTTMRLALFSVLTLLSFHAVVSVTPLHVQHPLDPLTKEEFLAVQTIVQNKYPISNNRLAFHYIGLDDPEKDHVLRYETHPTLVSIPRKIFVVAIINSQTHEILINLRIRSIVSDNIHNGYGFPILSVDEQSLAIKLPLKYPPFIDSVKKRGLNLSEIVCSSFTMGWFGEEKNVRTVRLDCFMKESTVNIYVRPITGITIVADLDLMKIVEYHDRDIEAVPTAENTEYQVSKQSPPFGPKQHSLTSHQPQGPGFQINGHSVSWANWKFHIGFDVRAGIVISLASIYDLEKHKSRRVLYKGYISELFVPYQDPTEEFYFKTFFDSGEFGFGLSTVSLIPNRDCPPHAQFIDTYVHSANGTPILLKNAICVFEQYGNIMWRHTENGIPNESIEESRTEVNLIVRTIVTVGNYDNVIDWEFKASGSIKPSIALSGILEIKGTNIKHKDEIKEDLHGKLVSANSIGIYHDHFYIYYLDFDIDGTHNSFEKTSLKTVRIKDGSSKRKSYWTTETQTAKTESDAKITIGLAPAELVVVNPNIKTAVGNEVGYRLIPAIPAHPLLTEDDYPQIRGAFTNYNVWVTAYNRTEKWAGGLYVDHSRGDDTLAVWTKQNREIVNKDIVMWHVVGIHHVPAQEDFPIMPLLSTSFELRPTNFFERNPVLKTLSPRDVAWPGCSN

At1g31710

MAPLHFTILILFSFVIVVSSSSFTPPRHPFDPLTETELKLVRTIINKSYPVGPNHKFTFQYVGLNEPNKSLVLSWYSSPNHTIKPPPRQAFVIARDNGKTREIVLDFSSRAIVSDKIHVGNGYPMLSNDEQEASTELVVKFKPFIDSVAKRGLNVSEIVFTTSTIGWYGETKAEAERVIRLMPFYLDGTVNMYLRPIEGMTIIVNLDEMKVSEFKDRSVVTMPIANGTEYRISKLNPPFGPTLHNAVLLQPDGPGFKVDGHIVRWANWEFHISFDVRAGIVISLASLFDTDVNKYRQVLYKGHLSEMFIPYMDPSDDWYFITYLDCGDFGCGQCAVSLQPYTDCPAGAVFMDGIFAGQDGTPAKIPKVMCIFEKYAGDIMWRHTEAEIPNLEITEVRPDVSLVARIVTTVGNYDYIVDYEFKPSGSIKMGVGLTGVLEVKPVEYIHTSEIKLGEDIHGTIVADNTVGVNHDHFVTFRLHLDIDGTENSFVRNELVTTRSPKSVNTPRKTYWTTKPKTAKTEAEARVKLGLKAEELVVVNPNRKTKHGNEVGYRLLHGSAAGPLLAQDDFPQIRAAFTNYNVWITPYNRSEVWAGGLYADRSQGDDTLAVWSQRNRKIEKEDIVMWYTVGFHHVPSQEDYPTMPTLSGGFELRPTNFFERNPVLKTKPVKVTTARKCTPKND

At1G31670

MVNRDNSIVALSFFMLFLLVLHLHFETTTAARKPVRVFGPPSSIEWSPPSPPKDDFEWFEINIYKNIEQTAFRPTGQGPSQGIGHKDPPVFSSVFVIGSLSFIPPPHPFDPLTEIELNLVRNIINERYPIGLEHRFTFQYVGLNEPDKSLVLSWVSSQYHNVKSPPRQAFVIARDHGKTREIVVDFASQAIVSEKIHVGNGYPMLTIDEQQATSELVLKFKPFRDSIRRRGLNVSEVVVTTSTMGWFGEAKPERLIKKRPFYLNGSVNTYLRPIEGMTIIVNLDQMKVTKFRDRFTSPLPNAKGTEFRISKLKPPFGPSLQNAVLFQSEGPGFKIDGHTNRWANWEFHMSFDVRAGLVISLASIFDMDVNKYRQVLYKGHLSEIFVPYMDPSEDWYFRTFFDCGEFGCGQYAVSLEPYTDCPGNAAFMDGVFASQDGTPIKITNVMCIFEKYAGDIMWRHTEIEIPGLKVRPDVSLVVRMVTTVGNYDYIVDYEFKPSGSIKIGVGLTGVLEVKPVKYVNTSEIKEDDIHGTIVADNTIGVNHDHFVTYRLDLDIDGTDNSFVRSELVTKRTPKSVNTPRKSYWTTKRLKAEELLVVNPSRKTKHGNEVGYRLLHGPASEGPLLAQDDYPQIRAAFTNYNVWITPYNNTEVWASGLYADRSQGDDTLAVWSQRNRKIEKTDIVMWYTVGFHHVPCQEDFPTMPTLFGGFELRPTNFFEQNPDLKTKPIKLNTTPTCTARND

At1G31690

MAQVHLTIFIFSSIFVISSSSFIPPPHPFDPLTETELKLVRNIINKSYPIGHNHKFTFQYVGLNEPEKSLVLSWHSSPDRNVKPPPRQAFVIARDKGMSREIVIDFSTRAIVSNKIHVGNGNPMLTIDEQQAATAVVQKYKPFCDSIIKRGLNLSEVVVTSSTMGWFGETKTKRFIRTIPFYLNGSVNTYLRPIEGMTIIVNLDQMKVTGFKDRFTGPMPKANGREYRISKLKPPFGPSLRSAVVFQPDGPGFKIDGHVVRWANWEFHMSFDVRAGLVISLASIFDMDMNRYRQVLYKGHLSEMFVPYMDPNDDWYFISYLDCGEFGCGQTAVSLEPYTDCPPNAAFMDGIFPGQDGTPTKISNVMCIFEKYAGDIMWRHTEAEVPGLKITEVRPDVSLVARMVTTVGNYDYIIEYEFKPSGSIKMGVGLTGVLEVKPVEYVHTSEIKEDDIYGTIVADNTVGVNHDHFVTFRLDLDIDGTENSFVRTELVTKRTPKSVNTPRKSYWTTKRNTAKTEADARVKLGLRAEELVVVNPTKKTKHGNEVGYRLLPGPASSPLLVQDDYPQIRAAFTNYNVWITPYNKSEVWASGLYADRSQGDDTLAVWSQRDREIENKDIVMWYTVGFHHVPCQEDFPTMPTMFGGFELRPTNFFEQNPVLKAKPFNLTTIPKCTTKNE

At4G14940

MNTSILAILFLIQCVFTLGLHFHPLDPLTPQEINKTSFIVKKSHLGNLKDLTFHYLDLEEPNKSHVLQWLSPNPSKKPPPPRRRSFVVVRAGGQTYELIIDLTTSKIASSRIYTGHGFPSFTFIELFKASKLPLTYPPFKKSILDRSLNISEVSCIPFTVGWYGETTTRRELKASCFYRDGSVNVFTRPIEGITVTIDVDSMQVIKYSDRFRKPIPDKEGNDFRTKHRPFPFFCNVSDTGFKILGNRVKWANWKFHVGFTARAGVTISTASVLDPRTKRFRRVMYRGHVSETFVPYMDPTYEWYYRTFMDIGEFGFGRSAVNLQPLIDCPQNAAFLDGHVAGPDGTAQKMTNVMCVFEKNGYGASFRHTEINVPGQVITSGEAEISLVVRMVATLGNYDYIVDWEFKKNGAIRVGVDLTGVLEVKATSYTSNDQITENVYGTLVAKNTIAVNHDHYLTYYLDLDVDGNGNSLVKAKLKTVRVTEVNKTSSRRKSYWTVVKETAKTEADGRVRLGSDPVELLIVNPNKKTKIGNTVGYRLIPEHLQATSLLTDDDYPELRAGYTKYPVWVTAYDRSERWAGGFYSDRSRGDDGLAVWSSRNREIENKDIVMWYNVGFHHIPYQEDFPVMPTLHGGFTLRPSNFFDNDPLIG

Solyc03g005160.2.1

MKFSMEFIKVLALPIVIIVFLFCFTRSQCHHPLDPLSPSEINKIRVIIQKSHFSSLSNLTFHFVDLEEPKKEDVLHWMSLHKHKHKHVVSPYRRARVVVRANSETYEVVVDLSTRAIISENVYTGHGYPPITLDDIIQSSRLTLRNHQLQDSILRRGLNISEVSCIPQPAGWFGELKTRRVLNVPCFYRRGTTNFWARPIEGITTLVDVESMKIIKYIDRFRVPLPKAESAIFESSSQGSVTCNDSDTSRIIIKGNEVKWANWDFHVGFNTRAGTIVSTASIFDTTIKKYRRVLYRGHVSETFVPYMDPTFEWYYRTFMDIGEFGFGRSASSLVPLLDCPSNAVYMVGYMADSEGGVVQVPNAICIFERYVGDAAWRHTENGVPGNTKSKGQQEVNLVVRMVATVGNYDYTLDWEFKQSGAIKVGVSLTGVMETKAVKYTNDNQITEDAYGTLVAENTIAVNHDHFLTYYLDVDVDGTDNSFIKSKLITTTVKERKVSPRKSYWKVVKETMKAESEAKTNLGQEPVELLIVNSNKKTKVGNDVSYRLIPSRPAMSLLSDDDYPQRRAAYMKYQLWVTPYNKSERWAGGFYTDRSHGDDGLAIWSLRNRTIKNKDIVFWYTVGLHHVPCQEDFPVMPSIYEGFELRPTNFFQRNPLLKQ

Solyc09g075940.2.1

MELTKVLHIFIAFFFLFFSINSQSHPLDPLNPDEINKIRVIIQKSHLSNVTFHDVDLDEPEKNDVLNWLSFHKSNNASFPYRRARVVVRANGESYELTLDLAASSIISQKLYSGHGFPRFTLNELRQAISLPLSSPEFQDSISRRGLNISEVSCIPLSVGWFGQAKTRRVLNVPCLYRGGTTNIWTRPIEGITILVDIESMKIIQYLDRFRAPLPEARDADFNSSSQGSVTCNETGSSRINIQGHEVSWANWKVHVGFNTRAGMIISTASIFDAVRNEYRRVLYRGHASEIFVPYMDPTFEWYYRTFFDIGEYGFGGSASTLVPSLDCPNNAVYMDGYMADSESQVVQIPRAICIFERYAGDAAWRHTENGNTKGQKEVNLVIRMVATVGNYDYILDWEFKQSGSIKVGASLTGIMAMKAVKFTNKDQINQDVYGTLVAENIVAVNHDHFLTYRIDLDVDGSKNSFLKAKLKTTRVKDQKTSPRKSYWSVVKETMKTESEGRTQIGTEAADLLFVNTVKKSKLGNEVGYKLIPSRPSISLLTDDDYPQIRAAYTKYQLWVTPYNKSERWAAGFYADRSHGDDGLTVWSGRNRSIEKEDIVLWYTLGFHHAPCQEDYPIMPTINDAFELRPTNFFERNPLLD

Solyc09g075930.1.1

MELIKVLHISFAFFFLFFSINCQSHPLDPLNPDEINKIRVIIQKSHLSNVTFHDVDLDEPEKNDVLHWLSNHKLHNASFPYRRARVVVRANGESYELTLDLAASSIISQKLFSGHGFPPFTSNEAIQSSILPLSNPKFQESISRRGLNISEVSCIPLSVGWFGQDKTIRVLSLPCFYSGGTTNIWARPIEGITILVDVESMKIIEYLDRFRTPLPEAKDADFNSSSQGSVTCNETGSSRIKIEGHEVSWANWKLHVGFNTRAGMIISTASIFDAVRSEYRRVLYRGHPSEIFVPYMDPTFEWYYRTFMDAGEYGFGRAASTLVKSLDCPNNAVSMDGYMADSESQVVQVPRAICIFERYAGNAAWRHTENENTKGQKEVNLVIRMVATVGNYDYILDWEFKQSGSIKVGASLTGIMEMKAVKYTNNEQINQDVYGTLVAENTIAVNHDHFLTYRIDLDVDGSKNSFLKAKLKTTRVKDQKMSPRKSYWSVVKETMKTESEGRTQLGIEAAELLFVNTEKKSKIGNEVGYKLIPSRPTMSLLTDDDYPQIRAAYTKYQLWVTPYNKSERWAAGFYADTSHGDDGLTVWSGRNRSIEKEDIVLWYTLGFHHVPCQEDYPIMPTLYDAFELRPTNFFERNPLLD

At1G62810

MAEPSFARLFLLFFSFLLIFATYSWVFGPDSGFLFGTRVRKTLGSNRQVHVDHSLEKPHHPLDPLTVREINRVRTILSNHDPGFGSGSATIHSMALDEPEKSRVVQWKKGNKLLSRRAAVVAYWGGQTHEITVDLDSGRVVSDVINRTSGYPILTLNDVFAASQVPLKSLEFNRSIEARGVKFSDLACITPFAGWFGSEEEGRRVIRVQCFTLQGTTNYFMRPLEGLYVTVDLDKLEVIKIIDKGPIPIPKASGTEYRFGVQNKPVHMDRINPISMEQPDGPSFRVEDGHLVKWANWVFHVKADQRAGMIISQATVRDSETGEPRSVMYKGFPSELFVPYMDPEEGWYYKGYMDAGELGLGPTAMPLVPLNDCPRNSYYIDGVFASPDGKPIVQPNMICLFERYAGDISWRHSEILFANADIRESRPKVTLVARMATSVGNYDYIFDWEFQTDGLIRVTVAASGMLMVKGTPYDNVDDLGDREDDAGPLISENVIGVVHDHFITFHLDMDIDGPMNNSLVKVHLEKQRVPTGKSPRKSYLKVKKYIAKTEKDAQIKLSLYDPYEFHIVNPNRKSRVGNPAGYRIVPGGNAASLLDHDDPPQIRGAFTNNQIWVTPYNRSEQYAGGVLIYQSQGDDTLQVWSDRDRSIENKDIVLWYTLGFHHVPCQEDYPVMPTVAASFELKPANFFESNPILGSAPFFEKDLPVCRPFASS

At3G43670

MVELSFSQLLVLLLSLLFLFTTLASSSKTPRFKYSLEKPHHPLDPLTTPEIKRVQTILSGHDPGFGSGSTIIHAMALDEPDKQRVIRWKKGDRLPPRRAEILAMSNGESHVLTVDLKSGRVVSDLVNPTFGYPILTMKDIIAVSQVPYKSVEFNRSIEARGIPFSGLICITPFAGWYGPDEEGRRVIKIQCFSKQDTVNFYMRPIEGLYLTVDMDKLEIIKIVDNGPVPVPKSTGTEYRYGFLNETVYMDRVNPMSMEQPDGPSFQVEDGYLVKWANWKFHIKPDQRAGMIISQATVRDSKTGEARSVMYKGFASELFVPNMDPGEGWYSKAYMDAGEFGLGPSSMPLVPLNDCPRNAYYIDGFFASPEGIPILQPNMICLFERYAGDTSWRHSEILLPGVDIRESRAKVTLVARMACSVGNYDYIFDWEFQMDGVIRVTVAASGMLMVKGTAYENVEDLGEKEDDSGPLISENVIGVVHDHFISFHLDMDIDGSANNSFVKVHLEKQRLPPGESRRKSYLKVKKYVAKTEKDAQIKMSLYDPYEFHLVNPNRLSRLGNPAGYKLVPGGNAASLLDHDDPPQMRGAFTNNQIWVTRYNRSEQWAGGLLMYQSRGEDTLQVWSDRDRSIENKDIVLWYTLGFHHVPCQEDFPVMPTIASSFELKPVNFFESNPVLGISPFFEKDLPVC

At4G12270

MEPKLSSRLFLLITTACFILLFTSTNFSQAPSSTAKLFDCTKSSSSPFCASRNFLFNKNTRPISKNIPKTKPKNHDHVSDTPNHPLDPLTVMEINKVRSILSSHALFASRVPHLLNSVVLEEPDKNLVRQWEKGDQLPPRKASVIARVGGNSHLLIVDLSTSRVDQADSPVPESGYPIVTSEEMDSAASAPFSNADFNRTINSRGVNLTDVICIPISSGWFGNKDDNTKRVTKIQCFSTQDTPNFYMRPIEGLTLLFDLDTKRILEITDTGQSIPIPGSTNTDYRYSTLPNHDKTRPLNTISLEQPRGPSFVIEDNHLVKWANWEFHLKPDPRAGLIMSQVKIHDPDTQETREVMYKGFVSELFVPYMDPSEAWYFKTYMDAGEYGFGLQAMPLEPVAEPKNLFIGPLNDCPRNAVYMDGTFAAADGTPYVRENMICVFESYAGDIAWRHTEYPVNGMPN

At4G12290

MDQKSFFRLIFLIVTAGFIISFTSTNFPHAPTRLLDCTDSSSSPLCASRNFLFNKQQPRPIPKHDPKPNTKNHDHVSDTPNHPLDPLTVSEINKIRSILSSHALFTSGTPHALHTVVLEEPEKNLVRHWEKGNPLPPRKASVIARVGADTHVLTVDISTGRVDSENSPVRVSGYPMMTIEEMNDITVVPFSNADFNRTIISRGVNLTDVICFPISCGWFGNKEENARVIKSQCFMTQGTPNFYMRPIEGLTILIDLDTKQVIEITDTGRAIPIPGSTNTDYRFQKLATTDKTRPLNPISIEQPRGPSFVIEDNHLVKWANWEFHLKPDPRAGVVISRVRVHDPDTHETRDVMYKGFVSELFVPYMDPSDAWYFKTYMDAGEYGFGLQAMPLVPLNDCPRNAAYMDGVFAAADGTPFVRENMVCIFESYAGDIGWRHSESPITGIPIREVRPKVTLVVRMAASVGNYDYIIDYEFQTDGLIKAKVGLSGILMVKGTTYQNKNQVEKDKDGNEEELHGTLLSENVIGVIHDHYVTFYLDLDVDGPDNSFVKVNLKRQETEPGESPRKSYLKAVRNIAKTEKDGQIKLSLYDPSEFHVINSGKTTRVGNPTGYKVVPRTTAASLLDHDDPPQKRGAFTNNQIWVTPYNKSEQWAGGLFTYQSHGDDTLAVWSDR

Solyc08g079430.2.1

MEGRNLFFLMSIILLLIFTFLNLPSPLSHTTDQLFDCTTYSPWCSSKKPFFLRQPENSKNHYADFPNHPLDPLTIGEIQKVKKIVHSIAEFRIKGCVLHSVVLEEPEKEVVLNWRRGHRLPPRKAAVVARALGVVHVLSVDIETGRVTRRETGSYSGYPILTIEDMISATSAPFANSDFNRSIIQRGVDLADLACLPVAAGWYGKIEEKRRVIKVQSFTLKDTINFYMRPIEGLTVLLDLDTQQVIEIFDEGESIPIPKAANTDYRYSRIKKNKQKINLLKPISIEQPNGPSFTIENNHLVKWANWEFHLKPDPRAGVIISRVMFEDPDTGKMRNVMYKGFTSELFVPYMDPSDAWYFKTYMDAGEYGFGLQAMPLDPLNDCPRNAYYMDGVFVAADGTPYVRSNMICVFERYAGDIGWRHAECPITGLPIREVRPKVTLVVRMAASVANYDYIVDWEFQNDGLIRPKVGLSGILMVKGSPYVNMNEVNQNEYLYGTLLSENIVGVIHDHYVTFHLDMDIDGPSNNSFVKVNLQKEMTSSGESPRRSYLKAVRNVAKTEKDAQIKLKLYDPSEFHVINSNKKSRVGNPVGYKVVPGGTAASLLDHNDPPQKRAAFTNNQIWVTPYNESEQWAAGLFVYQSQGDDTLAVWSDRDRAIENKDIVLWYTLGFHHIPCQEDFPIMPTVSSSFEIKPVNFFESNPILNIPPNSPKDLPICKAAASA

NtCuAO3/KJ730259.1

MEGRNFLRYLFFLMSIILLLFFTFLNLPSPLSHTTELLDCTTYSPWCSSRKPFFLRNSNKDKKHYADFPNHPLDPLTIQEIQKVKKIINSLDDFKVKGSYVLHSVVLEEPEKEVVLNWRKGQRLPSRKASVVARAHGDVHVLTVDIEADRVTHREMDHLSGYPILTIEDMITATAAPLGNSDFNRTIIQRGVDLGDLACLPISTGWYGKVEEKRRVIKVQCYTMKDTINFYMRPIEGLTVLLDLDTKQVIEILDEGKNIPIPKAADTDYRFSTQKNTQALNLLKPISIEQPNGPSFTIEDNHIVKWANWEFHLKPDPRAGVIISRVMVQDPDTGKMRNVMYKGFTSELFVPYMDPSDAWYFKTYMDAGEYGFGLQAMPLDPLNDCPRNAYYMDGVFVAADGTPYVRSNMICVFESYAGDIGWRHAESPITGLPIREVRPKVTLVVRMAASVANYDYIVDWEFQTDGLIRVKVGLSGILMVKGSPYVNMNQVNQNEYLYGTLLSENIIGVIHDHYITFHLDMDIDGSNNSFVKVNLQKAMTSPGESPRKSYLKAVRNVAKTEKDAQIKLKLYDPSEFHVINPNKKSRVGNPVGYKIVPGGTAASLLDLDDPPQKRGAFTNNQIWVTPYNESEQWAAGLFVYQSQGDDTLAVWSNRDRPIENKDIVLWYTLGFHHIPCQEDFPIMPTVSSSFEIKPVNFFESNPILXIPPNSPNDLPICKAAASA

HiCuAO4

MVEVSEHTASLTTNPPKKRKKDMEAKLILPSSSLFLFFILLLVLFTLSTIPFAPPSNKAELLDCSIYSPWCTSKNRIQSKNHPPTRHHHSSSDVVPHHPLDPLTIQELNRVRDIIKSHALFNNSPYALHSVVVEEPDKPVVLAWTKGDPLPRRKASVVARVNGVSHVLSVDLANGEVTRGQEVDQLSGYPTMTMEDMTSSTWAPLADADFNRTITQRGVDLADLACLPISTGWFGKKEDNRRLIKVQCYSMKDTANFYMRPIEGLTVVLDMDTKEVLEISDRGSNIPIPKAANTDYRYSVQNAHQLKLVNPISIEQPEGASFTIQDEHLVKWANWEFHLKPDPRAGVIISSAKVQDPETGKFRNVMYKGFTSELFVPYMDPTDAWYFKTYMDAGEYGFGLQAMPLDPLNDCPRNAYYMDGVFAAADGTPYVRSNMVCVFESYAGDIGWRHSESPITGMGIREVRPKVTLVVRMAASVANYDYIVDWEFQMDGLIRVKVGLSGILMVKGTSYINMNQVNKQESLYGTLLSENVIGVIHDHYVTFHLDMDVDGIDNSFVKVNLQKEHPSPEESPRRSYLKAVRNVAKTEKDAQVKLKLYDPSEFHVVNPSKLTRVGNPVGYKVVPGGTAASLLDLNDPPQTRGAFTNNQIWVSPYNKSEQWAGGLFTYQSHGEDTLAQWSDRDRPIENKDIVVWYTLGFHHIPCQEDFPIMPTVSSSFDIKPVNFFESNPILRIPPNLEKDLPICKAAASA

Leryth_003263-t1

MQGKNLFGSLFFALSIVFLIYFTLFTSPYPPPDKAELLNCAAYSPWCTTSNHLRRGPRSPTRHLHSSSTPHHPLDPLTIKEINKVQAILKSHELFKNQVYAIHSLVLEEPEKSLVLAWNKGDELPRRKASVIARAKDVSHVLTVDIETSEVVEHDMGRVSGYPEMTIEDMTSSTWAPLANAEFNRSIVERGVDLADLACLPISTGWFGKKEDNRRLVKVQCYSMKDTANFYMRPIEGLTVVLDIDTKEVIEISDKGKNIPIPKAKNTDYTFSAQNGDQLKLVNPISLEQPQGPSFTVEDEHLVKWANWEFHLKPDPRAGVIISRAMVRDPDTGKLRNVMYKGFSSELFVPYMDPTDAWYFKTYMDAGEYGFGLQAMSLVPLNDCPRNAYYMDGVFTAADGTPFVRSNMICVFESYAGDIGWRHSESPITGMGIREVRPKVTLVVRMAASVANYDYIVDWVFQTDGLIRIKVGLSGILMVKGTSYVNMNQVNQEQSLYGTLLSENVIGVIHDHFVTFYLDMDVDGSENSFVKVNLQKEHPSPGESPRRSYLKAVRNVAKTEKDAQIKIKLYDPSEFHVINPSKKSRVGNPVGYKVVPAGTAASLLDHDDPPQRRGAFSNNQIWVTPYNQSEQWAGGLFVYQSHGDDTLAVWSDRDRPIENKDIVLWYTLGFHHIPCQEDFPIMPTVSSSFDIKPVNFFESNPILRTPPNMEKDLPNCRATASA

SoCuAO4

MQGITLFSCSFFTLTIIFLIHYTFFNLPSPPPDKAALLDCPAFSPWCNSNTLLRRGPKNPTRHLHSSAVPHHPLDPLTIKEINKVQAVVKSHALFHNQIYAIHSLVLEEPEKSVVLAWKKGDILPPRKASVIARVKDVSHVLTVDIGTSDVEVHEMSRVSGYPALTIEDMISSIRAPLSNEEFNRTIIERGVDLADLACLPISTGWFGKREDNRRLIKVQCYSMKDTANFYMRPIEGLTVVLDIDTKEVIEIPDKGKDIPIPKAKNTDYTFPAQNGHQLKLVNPISLEQPQGPSFTVEDEHLVKWANLEFHLKPDQRAGVIISRAMVRDPDTGKLRNVMYKGFSSELFVPYMDPTDAWYFKTYMDAGEYGFGLQAMSLVPLNDCPRNAYYMDGVFTAADGTPFVRSNMVCVFESYSGDIGWRRSESPITGMGIREVRPKVTLVVRMAASVANYDYIVDWEFQTDGLIRIKVGLSGILMVKGTSYVNMNQVNQEESLYGTLLSENVIGVIHDHYVTFYLDMDVDGSENSFVKVNLQKEHPSPDESPRRSYLKAVRNVAKTEKDAQIKLKLYDPSEFHVINPSKKSRVGNPVGYKVAPGGTAASLLDLDDPPQRRGAFSNNQIWVTPYNQSEQWAGGLFVYQSHGDDTLAVWSDRDRPIENKDIVLWYTLGFHHIPCQEDFPIMPTVSSSFDIKPVNFFESNPILRIPPSMEKDLPNCRAAYSA
