# Supplementary material for: Roots of Indian heliotrope (Heliotropium indicum) produce simple pyrrolizidine alkaloids using the same homospermidine oxidase involved in biosynthesis of complex pyrrolizidine alkaloids in aerial parts
Source: Plant Biol (Stuttg). 2025 Jul 18;27(7):1378–90. doi: 10.1111/plb.70077 (PMC12631511; doi:10.1111/plb.70077)
Supplement: Supplementary file 3 [file PLB-27-1378-s002.odt]

Table S1 Primer Sequences 

| No. | Primer sequence (5’ to 3’) | Purpose |
| --- | --- | --- |
| P1 | ATTGCACTCGACGGAAATACAGTG | N20 oligos for motif in exon 1 of hicuao1 gene, overhang for ligation underlined, Construct A |
| P2 | AAACCACTGTATTTCCGTCGAGTG | N20 oligos for motif in exon 1 of hicuao1 gene, overhang for ligation underlined, Construct A |
| P3 | ATTGAGCATCGATTTATGACCCCG | N20 oligos for motif in exon 2 of hicuao1 gene, overhang for ligation underlined, Construct A |
| P4 | AAACCGGGGTCATAAATCGATGCT | N20 oligos for motif in exon 2 of hicuao1 gene, overhang for ligation underlined, Construct A |
| P5 | ATTGTGGTTATCCGATGCTCACCG | N20 oligos for motif in exon 1 of hicuao3 gene, overhang for ligation underlined, Construct B |
| P6 | AAACCGGTGAGCATCGGATAACCA | N20 oligos for motif in exon 1 of hicuao3 gene, overhang for ligation underlined, Construct B |
| P7 | ATTGGGTACCGGAAACAAGCCTTG | N20 oligos for motif in exon 3 of hicuao3 gene, overhang for ligation underlined, Construct B |
| P8 | AAACCAAGGCTTGTTTCCGGTACC | N20 oligos for motif in exon 3 of hicuao3 gene, overhang for ligation underlined, Construct B |
| P9 | ATTGGCTATGCTGGAAATACACTG | N20 oligos for motif in exon 2 of hicuao5 gene, overhang for ligation underlined, Construct C |
| P10 | AAACCAGTGTATTTCCAGCATAGC | N20 oligos for motif in exon 2 of hicuao5 gene, overhang for ligation underlined, Construct C |
| P11 | ATTGGGCGGATAAATACACCCACG | N20 oligos for motif in exon 4 of hicuao5 gene, overhang for ligation underlined, Construct C |
| P12 | AAACCGTGGGTGTATTTATCCGCC | N20 oligos for motif in exon 4 of hicuao5 gene, overhang for ligation underlined, Construct C |
| P13 | GGGGACAAGTTTGTACAAAAAAGCAGGCTCCACCATGAATTCACCTCTTAAATTTCTCCTC                                               attB1                           Kozac    Start          Binding region | full-length cDNA hiCuAO1, genomic DNA encoding encoding hicuao1, in planta expression hiCuAO1 for, sequencing of genomic region in exon 1 of hicuao1 gene to test for mutations |
| P14 | GGGGACCACTTTGTACAAGAAAGCTGGGTCTTATTTTTCGAACTGCGGGTGGCTCCAGCCGCC                                          attB2                                  Stop                              Strep-Tag-II                  Linker CTCCACTTCACTGGTTGATTG                Binding region | full-length cDNA hiCuAO1, genomic DNA encoding encoding hicuao1, in planta expression hiCuAO1 rev |
| P15 | GGGGACAAGTTTGTACAAAAAAGCAGGCTCCACCATGGCCTCAACTTCGGAAAAAGCGA                                               attB1                           Kozac    Start          Binding region | full-length cDNA hiCuAO2, genomic DNA encoding encoding hicuao2, in planta expression hiCuAO2 for |
| P16 | GGGGACCACTTTGTACAAGAAAGCTGGGTCTTATTTTTCGAACTGCGGGTGGCTCCAAGCGCT                                          attB2                                  Stop                              Strep-Tag-II                  Linker GAGCTTGGCGATTAAACTTGCACC                Binding region | full-length cDNA hiCuAO2, genomic DNA encoding encoding hicuao2, in planta expression hiCuAO2 rev |
| P17 | GGGGACAAGTTTGTACAAAAAAGCAGGCTCCACCATGGCTTCAACTCGAAACCTTTTCT                                               attB1                           Kozac    Start          Binding region | full-length cDNA hiCuAO3, genomic DNA encoding encoding hicuao3, in planta expression hiCuAO3 for, sequencing of genomic region in exon 1 of hicuao3 gene to test for mutations |
| P18 | GGGGACCACTTTGTACAAGAAAGCTGGGTCTTATTTTTCGAACTGCGGGTGGCTCCAAGCGCT                                          attB2                                  Stop                              Strep-Tag-II                  Linker CTTGGCTTCGCTAGTTGATTTTGC                Binding region | full-length cDNA hiCuAO3, genomic DNA encoding encoding hicuao3, in planta expression hiCuAO3 rev |
| P19 | GGGGACAAGTTTGTACAAAAAAGCAGGCTCCACCATGGTTGAGGTCAGTGAGCACACCG                                               attB1                           Kozac    Start          Binding region | full-length cDNA hiCuAO4, genomic DNA encoding encoding hicuao4, in planta expression hiCuAO4 for |
| P20 | GGGGACCACTTTGTACAAGAAAGCTGGGTCTTATTTTTCGAACTGCGGGTGGCTCCAAGCGCT                                          attB2                                  Stop                              Strep-Tag-II                  Linker AGCAGAAGCAGCAGCTTTACAAATT                Binding region | full-length cDNA hiCuAO4, genomic DNA encoding encoding hicuao4, in planta expression hiCuAO4 rev |
| P21 | GGGGACAAGTTTGTACAAAAAAGCAGGCTCCACCATGGCTTCATCTCTCAACCTTCTCT                                               attB1                           Kozac    Start          Binding region | full-length cDNA hiCuAO5, genomic DNA encoding encoding hicuao5, in planta expression hiCuAO5 for |
| P22 | GGGGACCACTTTGTACAAGAAAGCTGGGTCTTATTTTTCGAACTGCGGGTGGCTCCAAGCGCT                                          attB2                                  Stop                              Strep-Tag-II                  Linker AGTCAAATTGACTTTCTTGGATGG                Binding region | full-length cDNA hiCuAO5, genomic DNA encoding encoding hicuao5, in planta expression hiCuAO5 rev |
| P23 | GGCGATTGTTCCTGTCCCAA | Sequencing of genomic region in exon 2 of hicuao1 gene to test for mutations |
| P24 | GCCCGGAAAATGCAGTATATAGG | Sequencing of genomic region in exon 3 of hicuao3 gene to test for mutations |
| P25 | CCATTGGGGTCAACTATTAGCATG | Sequencing of genomic region in exon 2 of hicuao5 gene to test for mutations |
| P26 | AACAACTGATTCCGGCACTAGTC | Sequencing of genomic region in exon 4 of hicuao5 gene to test for mutations |
| P27 | GGCGATTGTTCCTGTCCCAA | Sequencing of genomic DNA encoding hicuao1 |
| P28 | ACTGTGAATGTAGTGATAAAGTATGTG | Sequencing of genomic DNA encoding hicuao2 |
| P29 | AGCTCTGGTGGGAAATACAATTTTC | Sequencing of genomic DNA encoding hicuao2 |
| P30 | CTGTCCCGTCCTGTTGACAATAC | Sequencing of genomic DNA encoding hicuao2 |
| P31 | TCCTGTAAGTTTGACTTCTGCTTC | Sequencing of genomic DNA encoding hicuao2 |
| P32 | AAAGCAAAAGGGACCGAGTATAG | Sequencing of genomic DNA encoding hicuao3 |
| P33 | ATAATGTTTGGGTCACACCAT | Sequencing of genomic DNA encoding hicuao3 |
| P34 | TCCTGAATTTCTACGTAGATCTTGAC | Sequencing of genomic DNA encoding hicuao3 |
| P35 | CAATGCCGGAGATGTCTTGTG | Sequencing of genomic DNA encoding hicuao3 |
| P36 | GTCGATTTAGCGGACTTAGCATG | Sequencing of genomic DNA encoding hicuao4 |
| P37 | GATGTAATCGTAGTTGGCGACTG | Sequencing of genomic DNA encoding hicuao4 |
| P38 | CCATTGGGGTCAACTATTAGCATG | Sequencing of genomic DNA encoding hicuao5 |
| P39 | GACTAGTGCCGGAATCAGTTGTT | Sequencing of genomic DNA encoding hicuao5 |
| P40 | AACAACTGATTCCGGCACTAGTC | Sequencing of genomic DNA encoding hicuao5 |
